# Polyoxometalate-Based Catalysts for CO_2_ Conversion

**DOI:** 10.3390/molecules24112069

**Published:** 2019-05-30

**Authors:** Yanwei Cao, Qiongyao Chen, Chaoren Shen, Lin He

**Affiliations:** 1 State Key Laboratory for Oxo Synthesis and Selective Oxidation, Suzhou Research Institute of LICP, Lanzhou Institute of Chemical Physics (LICP), Chinese Academy of Sciences, Lanzhou 730000, China; cyw929@licp.cas.cn (Y.C.); chenqiongyao17@mails.ucas.edu.cn (Q.C.); 2 University of Chinese Academy of Sciences, Beijing 100049, China

**Keywords:** polyoxometalates, catalysis, carbon dioxide, photo/eletroreduction, carbonyl source

## Abstract

Polyoxometalates (POMs) are a diverse class of anionic metal-oxo clusters with intriguing chemical and physical properties. Owing to unrivaled versatility and structural variation, POMs have been extensively utilized for catalysis for a plethora of reactions. In this focused review, the applications of POMs as promising catalysts or co-catalysts for CO_2_ conversion, including CO_2_ photo/electro reduction and CO_2_ as a carbonyl source for the carbonylation process are summarized. A brief perspective on the potentiality in this field is proposed.

## 1. Introduction

As the main greenhouse gas produced by human activity, about 10 ± 0.5 gigatons CO_2_ were released in 2018, bringing the atmospheric CO_2_ concentration level over the threshold of 400 ppm [1]. The large increase of global CO_2_ levels has led to serious environment problem. Meanwhile, it is also the most abundant and nontoxic carbon resource for preparing useful compounds [2]. From a practical point of view, its catalytic conversion is of significance to the supply of renewable energy, chemicals, and mitigation of global warming.

**Figure 1:**
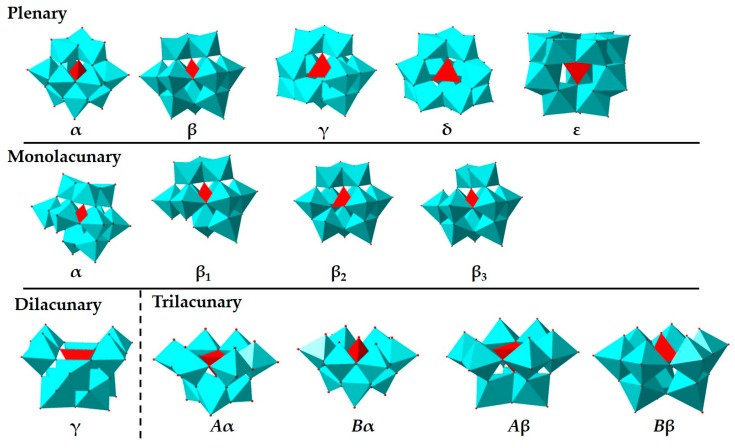
Isomers and mono- to trilacunary derivatives of the Keggin structure; color code: MO_6_ octahedron, light blue; XO_4_ tetrahedron, red (X = central atom).

Polyoxometalates with {MO_x_} (x = 5, 6) as basic construction units have derived an enormous fraternity of inorganic molecular complexes [3,4,5,6]. To obtain various composite materials with specific function, the modification and decoration of POM can be attained by partially substituting {MO_x_} units with different transition metal moieties or attaching organometallic complexes onto POM. Based on geometrical morphology, most of POMs can be classified as Keggin, Wells-Dawson, Anderson-Evans, Silverton, Waugh, Strandberg, Lindqvist, and Peacock-Weakley type structures, which are named after their corresponding discoverers [7]. Among these types, Keggin-type POMs ([XM_12_O_40_]^n−^) are comparatively well-studied. POMs discussed in the following sections are mostly Keggin type [8]. The original Keggin structure is designated α-, which consists of a tetrahedron central ion, [XO_4_]^n − 8^, caged by twelve MO_6_ octahedron. The Keggin structure includes four additional isomers (β-, γ-, δ-, and ε-), each resulting from 60° rotations of the four {M_3_O_13_} units [9]. The lacunary species of Keggin-type POMs stem from removing a variable number of {MO_6_} octahedra from the plenary polyanion, resulting in metal-oxide clusters with vacant addenda metal sites. Such vacant sites can be treated as inorganic multidentate ligands (Figure 1) [10], For example, sandwich-type POMs are usually synthesized by the reaction of transition metal ions with appropriate lacunary POM precursors [5,8]. The extraordinary diversity and synthetic accessibility of POMs have led to a wide spectrum of applications, ranging from catalysis, biochemistry/medicinal chemistry, to materials science [11,12,13,14]. In the field of catalysis, the development of POMs as oxidation and acid catalysts was flourish in the past few decades, with molybdic and vanadomolybdic clusters being more employed in the former case and tungstic ones in the latter [12,15]. The explorations of POMs as catalysts (or co-catalysts) for many other industrially important transformations were sought-after.

**Figure 2:**
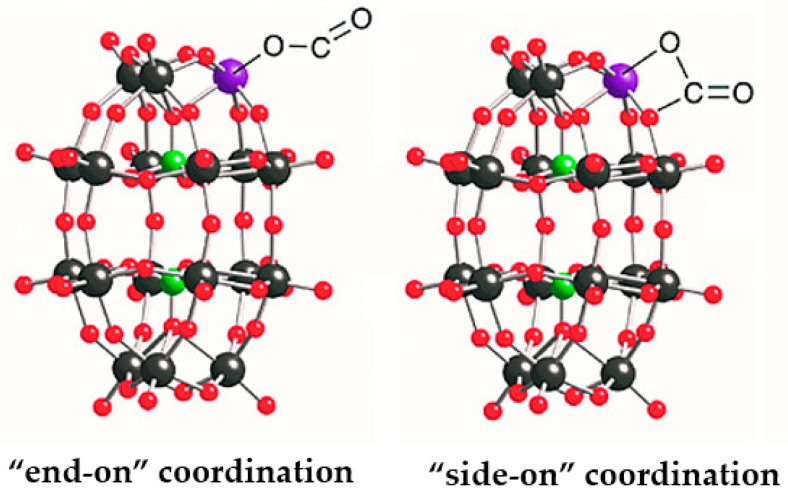
Suggested modes of coordination of CO_2_. Color code: W, black ball; P, green ball; O, red ball; Zn, purple ball. Reprinted with permission from [21]. Copyright © 2018 Wiley-VCH Verlag GmbH and Co. KGaA.

As early as 1988, Kozik and co-workers first reported the coordination of CO_2_ with several POM derivatives [16]. Afterwards more researches about the interaction of POMs with CO_2_ emerged [17,18,19,20,21,22]. Since the interaction between CO_2_ and POM is one of the crucial steps for catalytic transformations of CO_2_, it is necessary to clarify the mode of such interaction. However, considering the facts that CO_2_ can be transformed to ${\mathrm{CO}}_{3}^{2-}$ or ${\mathrm{HCO}}_{3}^{-}$ with water and this transformation is both reversible and temperature-dependent, the explicit “real” form of dissolved CO_2_ to interact with POM catalyst, was challenging to be justified. POMs displayed variable interacting modes. ^13^C NMR, UV/vis, IR with isotope-labelled CO_2_ (^13^CO_2_ and C^18^O_2_), and X-ray crystallography are useful tools to solve this issue. As pointed in the seminal report of Kozik et al. [16], the IR spectra and thermochromic behavior of POM α-[SiW_11_O_39_Co]^6−^ in the presence of either ${\mathrm{CO}}_{3}^{2-}$ or ${\mathrm{HCO}}_{3}^{-}$ was in contrast to the scenario with CO_2_. On ^13^C NMR spectra, the ^13^C chemical shifts of aqueous CO_2_, ${\mathrm{HCO}}_{3}^{-}$, and ${\mathrm{CO}}_{3}^{2-}$ are respectively at 125, 160, and 162 ppm. After bubbling CO_2_ into hydrous toluene solution of α-[SiW_11_O_39_Co]^6−^, the appearance of two signals at 792 and 596 ppm demonstrated the presence of two different kinds of paramagnetic CO_2_ species. In their report, two patterns were suggested on the basis of all ^13^C NMR, IR observations: (i) the complexation between α-[SiW_11_O_39_Co]^6−^ and CO_2_ was either via CO_2_ complexes with a direct η^1^ metal-carbon bond or bicarbonate complexes; (ii) the existence of H-bonding in the CO_2_ complexes was plausible. Other modes of POM-CO_2_ or POM-${\mathrm{CO}}_{3}^{2-}$ interaction were also discovered. Hill et al. reported the sandwich-type encapsulation of ${\mathrm{CO}}_{3}^{2-}$ via forming [(YOH_2_)_3_(CO_3_)(*A*-α-PW_9_O_34_)_2_]^11−^ [20]. [SiMo_11_CoO_38_(CO_2_)]_n_ polymeric chains reported by Xu et al. displayed the μ-η^1^,η^1^-OCO linear coordination mode of POM-CO_2_ interaction [21]. Two kinds of POM-CO_2_ interaction modes were observed on [(*n*-C_8_H_17_)_4_N]_8_[α_2_-P_2_W_17_O_61_Zn-(CO_2_)] [21]. The stronger “side-on” binding of CO_2_ by is predominant at higher temperatures (room temperature down to ca. 250 K) and the more weakly coordinated “end-on” POM-Zn-O-C-O structures were observable at lower temperatures (Figure 2). One particular case was uptaking 30 CO_2_ molecules by one capsule of [{(Mo^VI^)${\mathrm{Mo}}_{5}^{\mathrm{VI}}$ O_21_(H_2_O)_6_}_12_{${\mathrm{Mo}}_{2}^{V}$ O_4_(CH_3_COO)}_30_]^42−^. In this case, CO_2_ reacted directly with the H_2_O ligands in {${\mathrm{Mo}}_{2}^{V}$ O_4_(H_2_O)_2_}^2+^ linkers to generate ${\mathrm{CO}}_{3}^{2-}$ at pH 7. Then the uptake of 30 CO_2_ molecules was realized via the fast exchange of acetate by carbonate ligands [22]. Above cases indicated the abundance of POM-CO_2_ interaction mode.

In this context, we have witnessed the leap in developing POMs-based catalysts for the photo- or electrocatalytic CO_2_ reduction and CO_2_ as C1 synthon in organic synthesis. Herein, in this review, we focused on the recent experimental and theoretical advances in CO_2_ transformation with POMs-based catalyst or co-catalyst. The key factors for high-efficiency POMs-catalyzed CO_2_ conversion are highlighted.

## 2. Photocatalytic CO_2_ Reduction

Only high-energy vacuum ultraviolet laser (<200 nm) is able to directly excite and split CO_2_ into CO and O fragments [23], therefore highly efficient photocatalyst is vital to motivate and accelerate photocatalytic CO_2_ reductions with low-energy common visible light (>380 nm). This reduction process usually involves multi-electron transfer and the final products are carbon monoxide (CO), formic acid (HCOOH), formaldehyde (HCHO), methanol (CH_3_OH), and methane (CH_4_) [24,25]. The corresponding redox potentials for the possible CO_2_ reduction at pH = 7 are listed in Table 1.

Since the pioneering application of Honda–Fujishima effect in TiO_2_-catalyzed CO_2_ photoreduction was reported [26], multitudinous different type of photocatalysts, including simple metal oxides [25], perovskite oxides [27,28], C_3_N_4_ [29], MOFs [30], conjugated polymers [31,32], and POMs [33,34] have been developed. Since the seminal study on the photochemistry of molybdates and tungstates for analytical purposes over half century ago [35,36], the photoredox chemistry of POMs has been verified for a long time. The metals in POMs are fully oxidized with d^0^ electron configuration. Light absorption is mainly attributed to O→M ligand to-metal charge transfer (LMCT) bands in the wide range of the electronic spectra. Consequently, an electron is promoted from a spin-paired, doubly occupied bonding orbital (HOMO) to an empty, antibonding orbital (LUMO), thus an oxo-centered radical is generated. This photo-excited POMs are more reactive both in oxidation and reduction than the non-excited species [37]. In the photocatalysis by POM with LMCT mode, forming lower-energy O→M LMCT transitions is vital to more efficiently absorb visible light. Substituting addenda centers with different metals has been well demonstrated as one of the effectual tactics to tune the photochemical properties of POMs and optimize their visible light absorption via narrowing the band gap between valence band and conduction band. For instance, Streb et al. discovered that the vanadium substitution effectively improved the visible light absorption and photocatalytic activity of molybdate [Mo_6_O_19_]^2−^ [38]. TD-DFT calculations disclosed the corresponding contribution of O→V LMCT on absorption profile in the visible range. The progress of such strategy on POM-catalyzed selective photooxidation driven by visible light has been included in the recent review [15]. Besides altering the addenda centers on POMs, various other strategies have also been established in order to foster POMs as effective photocatalyst and most of their utilization were focused on photooxidation of water, photodegradation of organic dyes, as well as H_2_ evolution [37,39]. Here the progress of photoreduction of CO_2_ with POMs-based catalyst was summarized. 

### 2.1. CO_2_ to CO

#### 2.1.1. Homogeneous Catalysts

**Figure 3:**
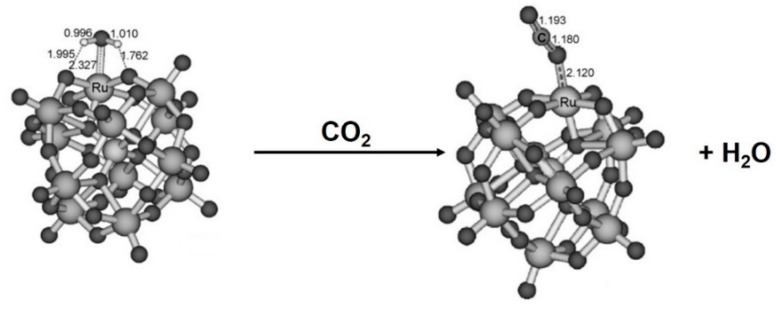
The substitution of H_2_O with CO_2_ in [Ru^III^(H_2_O)SiW_11_O_39_]^5−^. Reprinted with permission from [40]. Copyright © 2010 Wiley-VCH Verlag GmbH and Co. KGaA.

Neumann and co-workers reported that Ru-substituted POM ([Ru^III^(H_2_O)SiW_11_O_39_]^5−^) in combination with tertiary amines exhibited photoactivity for CO_2_ reduction Figure 3 [40]. Triethyl amine acted as the sacrificial agent. CO was the major product (~50 μmol after 20 h irradiation with ca. 2% quantum yield). None of HCOOH, MeOH, and CH_4_ was detected. UV/Vis, EPR, ^13^C NMR, and isotope labelling demonstrated that coordinated CO_2_ ([Ru^III^(CO_2_)SiW_11_O_39_]^5−^) was easily formed by substituting H_2_O in [Ru^III^(H_2_O)SiW_11_O_39_]^5−^. DFT results demonstrated that CO_2_ tended to coordinate to Ru^III^ by forming a Ru-O bond in an “end-on” manner. Based on these experimental and computational results, the authors concluded that the Ru^III^ site was responsible for activating CO_2_ via the coordination and the lacunary [SiW_11_O_39_]^8−^ site acted as photocatalyst in this process.

**Scheme 1:**
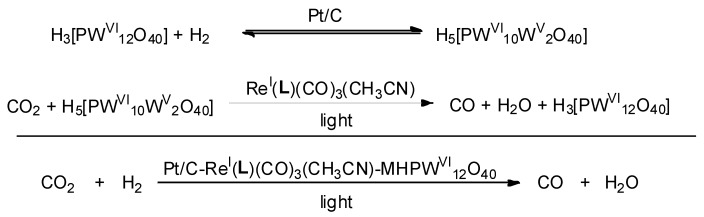
Proposed photoreduction of CO_2_ with H_2_.

**Figure 4:**
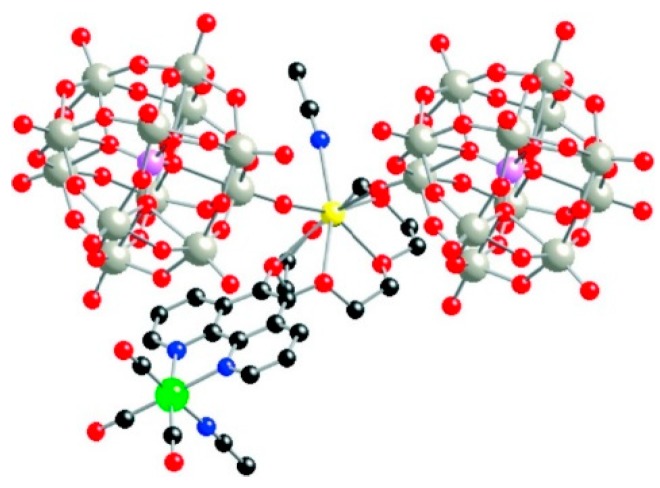
Re^I^(L)(CO)_3_-NaHPW_12_O_40_ presenting the complexation of two [PW_12_O_40_]^3−^ units to Na. H atoms and solvent molecules are omitted. C, black ball; N, blue ball; O, red ball; P, purple ball; Na, yellow ball; Re, green ball; W, gray ball. Reprinted with permission from [41]. Copyright© 2011 American Chemical Society.

By employing H_2_ instead of tertiary amines as the sacrificial agent, a viable CO_2_ reduction approach with Pt/C and Re^I^(**L**)(CO)_3_-${\mathrm{MHPW}}_{12}^{\mathrm{VI}}$ O_40_ (**L** = 5,6-(15-crown-5)-1,10-phenanthroline) as synergistic catalysts was developed. The grafting of Re^I^(L)(CO)_3_ complexes onto POMs was achieved via the complexation of crown ether moiety on **L** with the sodium cation binding two PW_12_O_40_ moieties (Figure 4) [41]. H_2_ was oxidized with the facilitation of Pt(0) on the Pt/C surface to afford two protons. In the following step, protons and electrons retained by Pt were simultaneously transferred onto Re^I^(**L**)(CO)_3_(CH_3_CN)-${\mathrm{MHPW}}_{12}^{\mathrm{VI}}$ O_40_ (${\mathrm{MHPW}}_{12}^{\mathrm{VI}}$ O_40_ + 2H^+^ + 2e^−^ → MH_3_ ${\mathrm{PW}}_{2}^{V}$ $W_{10}^{\mathrm{VI}}$ O_40_) (Scheme 1). Re^I^(**L**)(CO)_3_-MH_3_${\mathrm{PW}}_{2}^{V}$ $W_{10}^{\mathrm{VI}}$ O_40_ possessed the photoreductive activity for converting CO_2_ to CO, because its transition to excited CO_2_-reduction active state was allowed by absorbing visible light. CO were almost exclusively produced with turnover of 22.6% and 1.1% quantum yield. Besides CO, only trace CH_4_ but no further CO reduction products (e.g., CH_3_OH, HCHO, and HCOOH) was detected on GC-MS after 14 h irradiation.

**Figure 5:**
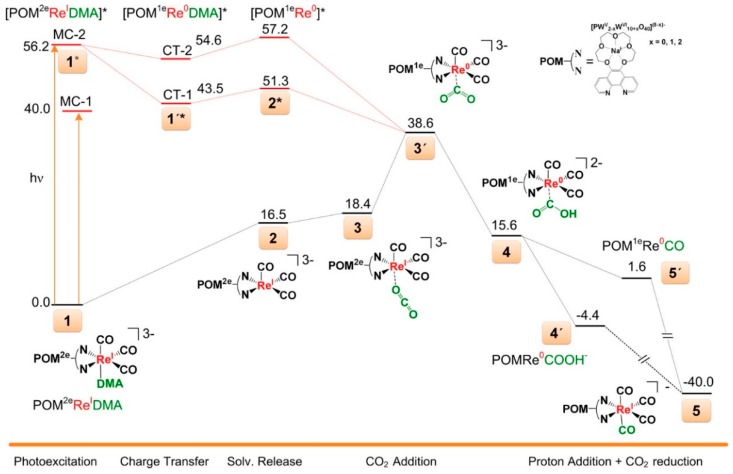
Potential energy (units in kcal mol^−1^) surface for the reduction of CO_2_ to CO catalyzed by POM-Re^I^ complex. Reprinted with permission from [42]. Copyright © 2016 American Chemical Society.

The TD-DFT calculations disclosed a more detailed mechanism for the photoreduction of CO_2_ to CO with this POM-Re^I^ complex catalyst [42]. Simulated absorption spectrum of Re^I^-POM complex was calculated at different functional level (i.e., X3LYP, M06X, B3LYP, and CAMB3LYP) and the computed results were found to be basically consistent with the experimental absorption spectrum (two major bands with maxima at 500 and 656 nm). In the ground state without any light irradiation, bonding CO_2_ with Re^I^ center to afford the reactive POM-Re^I^-${\mathrm{CO}}_{2}^{-}$ complex had to overcome a high energy barrier (≥38 kcal mol^−1^), therefore, the direct CO_2_ reduction with Re^I^-POM occurred less likely (Figure 5). In the conditions with photoexcitation, the excited Re^I^-POM was allowed to interact with CO_2_ to form POM-Re^I^-${\mathrm{CO}}_{2}^{-}$. The subsequently protonation and further electron reduction process were exothermic by more than 78 kcal mol^−1^. These energy profiles revealed that the role of POM as photosensitizer in the reduced state, electron “shuttles” as well as electron/proton reservoirs in this process. Plausible CO_2_ photoreduction mechanism consists of five steps: (1) photoexcitation and charge transfer; (2) disassociation of solvent molecule; (3) CO_2_ coordination; (4) proton and electron transfer; and (5) release of CO and recoordination of solvent.

Recently, by replacing crown ether-affiliated 1,10-phenanthroline with 6-hydrazinyl-2,2′-bipyridine on the Re complex, the Re_2_(CO)_6_Cl_2_L_2_-H_3_PW_12_O_40_ (L = 6-hydrazinyl-2,2′-bipyridine) hybrid pair and related relay process for CO_2_ reduction by photocatalysis and electrocatalysis was developed [43]. Initially, [${\mathrm{PW}}_{12}^{\mathrm{VI}}$ O_40_]^3−^ was reduced to [${\mathrm{PW}}_{2}^{V}$ $W_{10}^{\mathrm{VI}}$ O_40_]^5−^ by two electrons at −1.302 V (versus Fc/Fc^+^, Fc= ferrocene). Then the reduced POM undertook the functions of photoreductant and electron/proton acceptors. Photo irradiation promoted the generation of Re^I^(L^−^)(CO)_3_ via the electron transfer from [${\mathrm{PW}}_{2}^{V}$ $W_{10}^{\mathrm{VI}}$ O_40_]^5−^ to Re^I^(L)(CO)_3_. This reductive Re^I^ intermediate possessed reactivity for the selective CO_2_ reduction. Only CO was observed in this catalytic electro-photochemical reduction process. The result in the dark conditions indicated that light facilitated the CO_2_ reduction. The formation of dirhenium complex-H_3_PW_12_O_40_ hybrid pair via acid-base interaction was indispensable to this catalytic electro/photochemical transformation.

#### 2.1.2. Heterogeneous Catalysts

**Figure 6:**
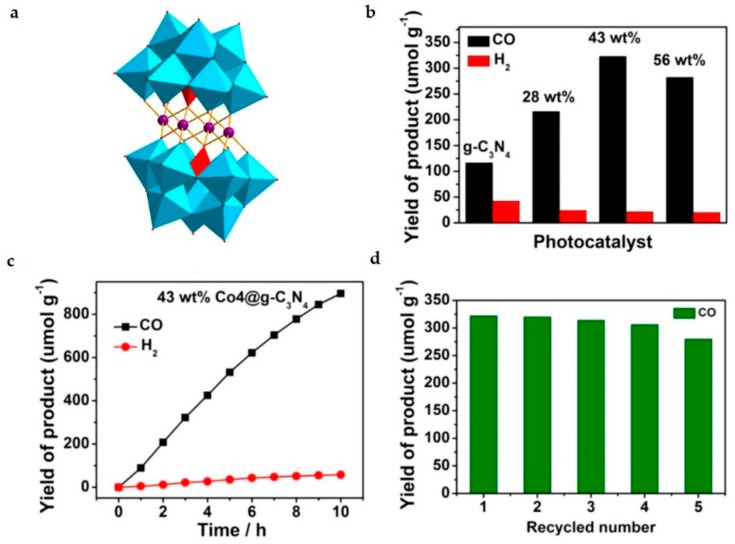
The photoreduction of CO_2_ by the Co_4_@g-C_3_N_4_ hybrid material. The structure of Na_10_[Co_4_(H_2_O)_2_(PW_9_O_34_)_2_]; color code: WO_6_ octahedron, light blue; PO_4_ tetrahedron, red; Co, purple ball; O, red ball. (**a**). The photocatalytic activity of different photocatalysts (**b**). Time course of the CO and H_2_ (**c**). Recycling experiments (**d**). Reprinted with permission from [44]. Copyright © 2017 American Chemical Society.

Photocatalytic CO_2_ reduction by POMs-based heterogeneous catalyst has received intensive research interests. As a POM-stabilized multi–Co-oxide clusters, [Co_4_(PW_9_O_34_)_2_]^10−^ is comprising a Co_4_O_4_ core stabilized by two oxidatively resistant polytungstate ligands (Figure 6a). The Na_10_[Co_4_(H_2_O)_2_(PW_9_O_34_)_2_ @graphitic carbon nitride hybrid material (Co_4_@*g*-C_3_N_4_) was reported for the efficient photocatalytic CO_2_ reduction [44]. It manifested the advantages of convenient recovery, steady reuse, simple preparation and flexible composition. The Co_4_@*g*-C_3_N_4_ photocatalyst with 43 wt% Co_4_ content showed high CO yield (107 μmol g^−^^1^ h^−^^1^) and excellent selectivity (94%) (Figure 6b). After 10 h reaction, the production of CO reached 896 μmol g^−^^1^ (Figure 6c). After 5 runs, the activity still remained (Figure 6d). Experimental and characterization results revealed that the Co_4_ unit both facilitated the charge transfer of *g*-C_3_N_4_ and significantly enhanced the surface catalytic oxidative activity.

**Figure 7:**
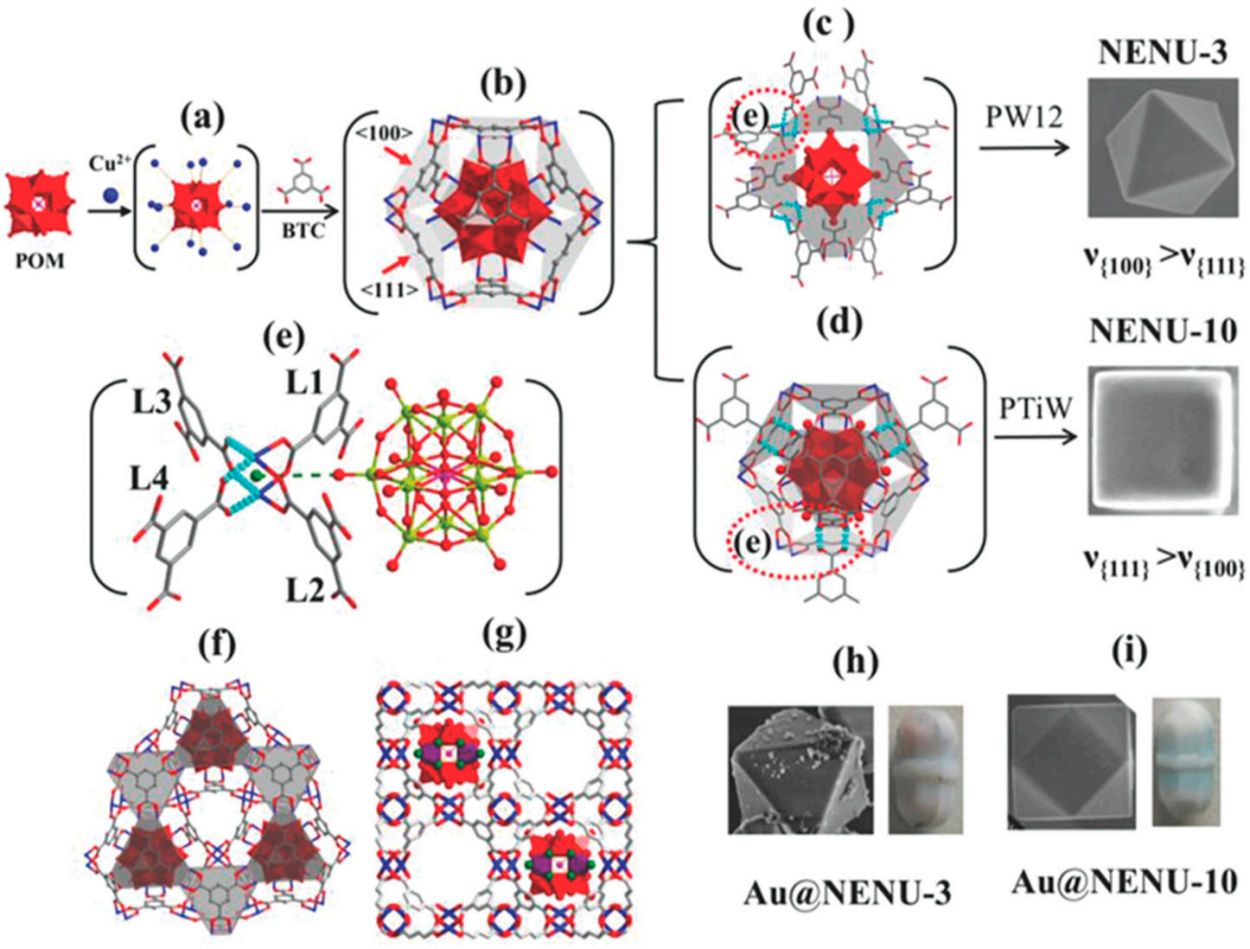
Proposed crystal growth mechanism of Au@NENU-3 and Au@NENU-10. The intermediate of Cu^2+^ around polyoxometalate (POM) (**a**). The proposed the basic growing unit consisting of 1 POM, 24 Cu^2+^, and 8 BTC ligands in which each Cu_2_ unit coordinates with 2 BTC ligands (**b**). Eight BTC ligands are needed to coordinate with four Cu_2_ units when crystal grows along the 〈100〉 direction (**c**). While only six BTC ligands are needed for three Cu_2_ units along the 〈111〉 direction (**d**). The enlarged diagram of the dashed red circle (**e**). The exposed surface of the octahedron shape for Au@NENU-3 ({111} plane where [PW_12_O_40_]^3−^ is sheltered by BTC (**f**). The exposed surface of the cube shape for NENU-10 ({100} plane where [PTi_2_W_10_O_40_]^7−^ is exposed wholly) (**g**). The SEM images for Au@NENU-3 and Au@NENU-10, respectively (h, i). Reprinted with permission from [45]. Copyright © 2018 Wiley-VCH Verlag GmbH and Co. KGaA.

NENU-10 and NENU-3 are respectively Ti-substituted Keggin-type POM [PTi_2_W_10_O_40_]^7−^ (This Ti-disubstituted Keggin-type POM contains the Ti centers in relative 1,5 positions according to the IUPAC nomenclature. This anion is one of the few cases in the literature in which a salt of a disubstituted Keggin species displays a predominant isomer (ca. 75% of the α (1,5) isomer)) and Keggin-type POM [PW_12_O_40_]^3−^ encaged into MOF Cu_3_(BTC)_2_ (BTC: benzene-1,3,5-tricarboxylate; Cu_3_(BTC)_2_ = HKUST-1) developed by Liu et al. Efficient photoreduction of CO_2_ catalyzed by hybrid material Au@NENU-10 (Both Au@NENU-10 and Au@NENU-3 were prepared by one-pot method. The Au NPs were in-situ deposited during the assembly of NENU-10 and NENU-3 as shown in Figure 7.) was recently achieved [45]. Table 2 illustrated the catalytic performance of as-prepared hybrid in the reductive CO_2_ transformation to CO and CH_4_ under visible-light irradiation. Compared to Ti-free Au@NENU-3, Au@NENU-10 presented both higher activity and selectivity in the CO_2_ photoreduction. Similarly, Au/K_7_(PTi_2_W_10_O_40_) also had better performance than Au/Na_3_(PW_12_O_40_). Results of control experiments implied the indispensability of Au nanoparticles to the CO reduction activity and precursors was inactive. Combing these patterns, authors deduced the following steps for the overall process: Firstly, Au nanoparticles produced electrons and holes where the holes oxidized H_2_O to generate two electrons and two protons. Then the electrons and protons directly transferred to [PTi_2_W_10_O_40_]^7−^ to form [P(Ti^III^-H)_2_ $W_{10}^{\mathrm{VI}}$ O_40_]^7−^. Some of [P(Ti^III^-H)_2_ $W_{10}^{\mathrm{VI}}$ O_40_]^7−^ species reduced CO_2_ to CO and H_2_, and others further obtained electrons and protons to form [P(Ti^III^-H_2_)_2_ $W_{2}^{V}$ $W_{8}^{\mathrm{VI}}$ O_40_]^7−^ intermediates. Finally, [P(Ti^III^-H_2_)_2_ $W_{2}^{V}$ $W_{8}^{\mathrm{VI}}$ O_40_]^7−^ reduced CO_2_ to CH_4_ and simultaneously returned to initial state. Ti-O-W in [PTi_2_W_10_O_40_]^7−^ may responsible to absorb CO_2_. Compared with [PTi_2_W_10_O_40_]^7−^ and [PW_12_O_40_]^3−^, [PTi_2_W_10_O_40_]^7−^ had stronger electron-coupling protons ability.

### 2.2. CO_2_ to HCOOH 

HCOOH is one of the promising hydrogen carriers and the important C1 source for organic synthesis. The generation of HCOOH in CO_2_ photoreduction consumes the same amount of protons and electrons as that for CO formation, but it requires slightly higher reduction potential.

**Figure 8:**
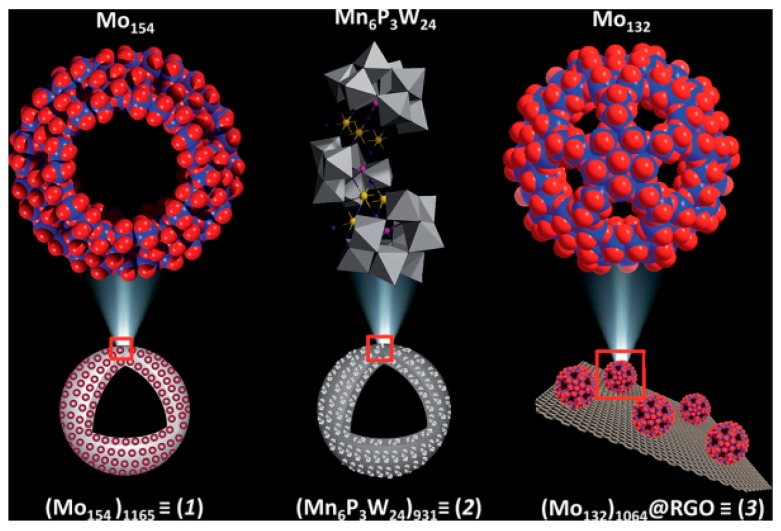
The structures of {Mo_154_}, {Mn_6_P_3_W_24_}, and {Mo_132_} POM macroions catalysts. Color code: Mo, red ball; O, blue ball; Mn, yellow ball; P, pink ball; WO_6_ octahedron, grey. Reprinted with permission from [46]. © 2016 Attribution-Non Commercial 3.0 Unported Licence (CC BY-NC 3.0).

The POM microions are the molecules denoted as {POM} in dilute solutions, they tend to afford suprestructural macroions denoted as {POM}_n_ (POM macroions). Three POM macroions catalysts, Na_15_[${\mathrm{Mo}}_{126}^{\mathrm{VI}}$ ${\mathrm{Mo}}_{28}^{V}$ O_462_H_14_(H_2_O)_70_]_0.5_[${\mathrm{Mo}}_{124}^{\mathrm{VI}}$ ${\mathrm{Mo}}_{28}^{V}$ O_457_H_14_(H_2_O)_68_]_0.5_ ({Mo_154_}_1156_), Na_17_[Mn_6_P_3_W_24_O_94_(H_2_O)_2_] ({Mn_6_P_3_W_24_}_931_) and (NH_4_)_42_[${\mathrm{Mo}}_{72}^{\mathrm{VI}}$ ${\mathrm{Mo}}_{60}^{V}$ O_372_(CH_3_COO)_30_(H_2_O)_72_]@RGO hybrid ({Mo_132_}_1064_@RGO; RGO = reduced graphene oxide) featured with peculiar structures, were respectively employed in the photocatalytic CO_2_ reduction coupling with water oxidation [46]. The enormous spherical superstructures in these three catalysts in dilute dispersion ({Mo_154_}_1156_, {Mn_6_P_3_W_24_}_931_, {Mo_132_}_1064_@RGO) are respectively made up by wheel-shaped {Mo_154_} rings, bent rod {Mn_6_P_3_W_24_} units and “Keplerate” {Mo_132_} spheres (Figure 8). The numbers of metal oxide cluster units in these superstructures were calculated by following equation: *n* = (4πR^2^)/(72.006σ^2^) × 60. (R, representing the hydrodynamic radius of POM vesicles formed in the dispersion, was obtained by dynamic light scattering analysis. σ, representing the diameter of isolated single cluster, was determined by the van der Waals radii of the constituent atoms.) The results showed that although HCOOH is the main product of CO_2_ reduction with these three catalysts, a considerable amount of HCHO was also obtained when {Mo_154_}_1156_ and {Mo_1__32_}_1064_@RGO catalysts were applied. In the case of {Mn_6_P_3_W_24_}_931_, CO_2_ was exclusively reduced to HCOOH. In terms of formic acid production, {Mo_154_}_1156_ and {Mo_132_}_1064_@RGO displayed better performance than {Mn_6_P_3_W_24_}_931_ (Table 3). This phenomenon can be rationally explained by that the simultaneous excitation of multitudinous photoactive clusters on the surface of {Mo_154_} and {Mo_132_} vesicles under light irradiation. For {Mo_132_}_1064_@RGO, good conductivity of RGO may help the facile electrons transfer.

Afterwards, POM macroions for the water oxidization-coupled CO_2_ photoreduction to HCOOH was extend to {Cu-PW_12_}_n=1348−2024_ ({Cu-PW_12_}_n_ = [(K_6.5_Cu(OH)_8.5_(H_2_O)_7.5_)_0.5_@(K_3_PW_12_O_40_)]_n_) [47] and gigantic oxo-molybdate catalyst Na_48_[H_x_Mo_368_O_1032_(H_2_O)_240_(SO_4_)_48_] ({Mo_368_}) [48]. In the former case, the max TON per mole was 613. Both FT-IR and Raman spectroscopies showed that the structure remained integral after reaction and the activity was kept after ten cycles. In the latter one, {Mo_368_}, which consisted of a central ball shaped unit {Mo_288_} and two {Mo_40_} capping units, showed excellent selectivity for HCOOH (95.73%) with impressive TON (27666) and TOF (4419 h^−1^). Its external quantum efficiency reached 0.6%. It is worth noting that in all above cases external photosensitizer was unessential. Because intervalence charge transfers (IVCT) of Mo^V^ to Mo^VI^ and W^V^ to W^VI^ have appropriate gap between conduction band and valence band, which can give rise to absorbance maxima in the region of visible light.

### 2.3. CO_2_ to CH_4_

Compared to the photoreductive conversion of CO_2_ to CO or HCOOH, photocatalytic methanation of CO_2_ involving an eight-electron transfer process was much more challenging. [PTi_2_W_10_O_40_]^7−^ was reported as the first POM-based photocatalyst for reducing CO_2_ to CH_4_ with CH_3_OH as electron donator [49]. Although [PTi_2_W_10_O_40_]^7−^ had poor activity and limited efficiency in photoreduction of CO_2_, this result suggested that POM could be an active catalyst in the CO_2_ photoreduction.

Since this time, few works focusing on photocatalytic CO_2_ reduction to CH_4_ during past decades. Recently, a remarkable achievement in photocatalytic CO_2_ reduction to CH_4_ with two hydrothermal-synthesized POM catalysts H[[Na_2_K_4_Mn_4_(PO_4_) (H_2_O)_4_]_3_[[Mo_6_O_12_(OH)_3_(HPO_4_)_3_(PO_4_)]_4_[Mn_6_(H_2_O)_4_]] (NENU-605) and H[[Na_6_CoMn_3_(PO_4_)(H_2_O)_4_]_3_[[Mo_6_O_12_(OH)_3_(HPO_4_)_3_(PO_4_)]_4_[Co_1.5_Mn_4.5_]] (NENU-606) was reported [50]. These two water-insoluble POMs showed good structural stability and extended solar spectrum absorption range in aqueous solutions.

**Figure 9:**
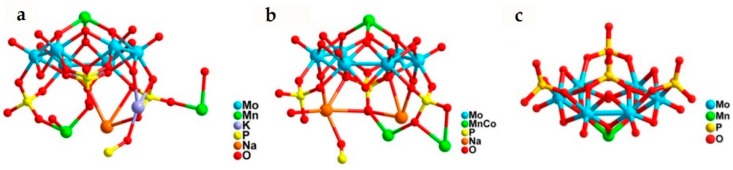
The asymmetric units of NENU-605 (**a**), NENU-606 (**b**) and NENU-607 (**c**). Reprinted with permission from [50]. © 2019 Attribution-Non Commercial 3.0 Unported Licence (CC BY-NC 3.0).

**Figure 10:**
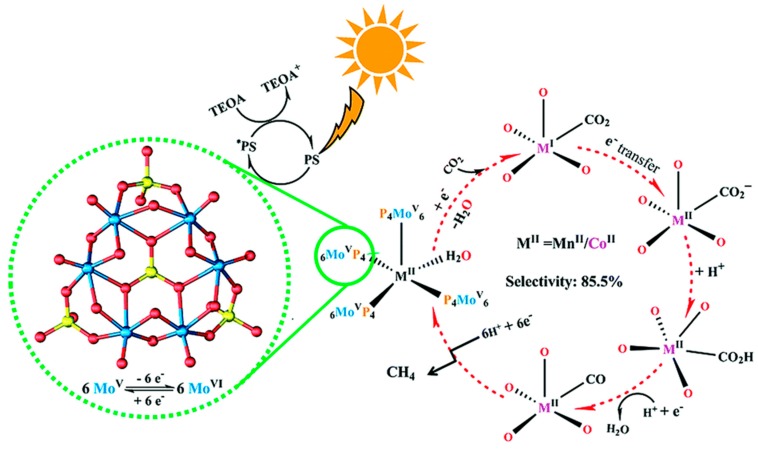
The proposed mechanism for photocatalytic reduction of CO_2_ to CH_4_ using NENU-605 or NENU-606. Color code: P, yellow ball; Mo, blue ball; O, red ball. Reprinted with permission from [50]. © 2019 Attribution-Non Commercial 3.0 Unported Licence (CC BY-NC 3.0).

Under CO_2_ atmosphere with triethanolamine (TEOA) as sacrificial agent and [Ru(bpy)_3_]Cl as photosensitizer, CH_4_ and CO were the main gaseous photoreduction products, only a trace amount of HCOOH was detected in the aqueous phase. The productivity of CH_4_ for NENU-605 and NENU-606 reached up to 170 nmol (894.7 nmol g^−1^ h^−1^) and 402 nmol (1747.8 nmol g^−1^ h^−1^) respectively. Good CH_4_ selectivity of 76.6% (NENU-605) and 85.5% (NENU-606) was achieved (Table 4, entry 1 and 2). Moreover, H_2_ evolution as the side reaction was not detected. Regarding to the higher CH_4_ selectivity of NENU-606 than NENU-605, the heterometallic Mn^II^/Co^II^ ions in NENU-606 might be more favorable to the adsorption and activation of CO_2_ than the homometallic Mn^II^ ions in NENU-605. In contrast, Mn[Mo_6_O_12_(OH)_3_(HPO_4_)_3_(PO_4_)]_2_ (NENU-607), the dimer containing only one Mn^II^ atom sandwiched between two P_4_Mo_6_ unit and displaying a similar connection mode to NENU-605 and NENU-606 (Figure 9), had much lower activity (Table 4, entry 3). Outcomes from control experiments demonstrated the necessities of both photosensitizer and light irradiation as well as the contribution from solvent effect (Table 4, entries 4–6). ^13^C-isotope labelling verified that CO_2_ was the carbon source of CO and CH_4_. Deduced from these patterns, a plausible multi-step mechanism for the photocatalytic CO_2_ methanation was proposed (Figure 10). Initially, the photosensitizer absorbs light to produce photo-excited electrons from its HOMO and then transfers electrons to the P_4_${\mathrm{Mo}}_{6}^{\mathrm{VI}}$ unit through the matched LUMO positions (P_4_${\mathrm{Mo}}_{6}^{\mathrm{VI}}$ + 6e^−^ → P_4_ ${\mathrm{Mo}}_{6}^{V}$). Simultaneously, the electron holes produced in the valence band of ruthenium complex was consumed by TEOA (TEOA + h^+^ → TEOA^+^). Subsequently, strongly reductive P_4_${\mathrm{Mo}}_{6}^{V}$ unit further transfer electrons to the active metal center (M^II^ + e^−^ → M^I^). Then the adsorbed CO_2_ (M^I^ → M^I^-CO_2_) obtains electrons from active metal sites (M^I^-CO_2_ → M^II^-${\mathrm{CO}}_{2}^{-}$) with the help of H_2_O as a proton source (M^II^-${\mathrm{CO}}_{2}^{-}$ + H^+^ → M^II^-COOH). M^II^-CO was formed via the proton- and electron-assisted dehydroxylation (M^II^-CO_2_H + H^+^ + e^−^ → M^II^-CO + H_2_O). CH_4_ is eventually produced with further six-electron transfer process (M^II^-CO + 6H^+^ + 6e^−^ → M^II^ + CH_4_ + H_2_O).

## 3. Electrocatalytic CO_2_ Reduction

Electrocatalytic CO_2_ reduction to produce various C1 and C2 molecules, for instance, CO, HCOOH, HCHO, CH_3_OH, CH_4_, oxalic acid, ethylene, and ethanol, is regarded as a promising proctol for the production of liquid fuels or bulk chemicals. The corresponding reactions and standard potentials were list in Table 5 [51].

Electrocatalytic reduction of CO_2_ with POM was first reported by Kozik et al. [52]. Cyclic voltammograms (CV) of several POMs with or without the presence of CO_2_ in various nonpolar solvents were compared. For α-[SiW_11_O_39_Co]^6−^, a large change on its CV was recorded after CO_2_ was bubbled. Meanwhile for α-[P_2_W_18_O_62_]^6−^, almost no change was observed before and after bubbling CO_2_. The evidence from CV hinted that α-[SiW_11_O_39_Co]^6−^ might be active to CO_2_ reduction. Further investigations on reduced α-[SiW_11_O_39_Co]^6−^ with CV indeed confirmed that α-[SiW_11_O_39_Co]^6−^ showed the electrocatalytic for CO_2_ reduction. However, the final CO_2_ reduction product was unstated.

**Figure 11:**
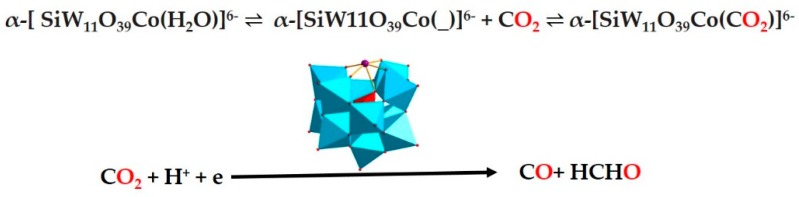
Electroreduction of CO_2_ by [SiW_11_O_39_Co]^6−^ catalyst. Color code: WO_6_ octahedron, light blue; SiO_4_ tetrahedron, red; Co, purple ball; O, red ball. The notation of (_) in the above figure represents a vacant coordination site.

More than decade later, Proust et al. reinvestigated this reaction in more detail by using [α-SiW_11_O_39_Co]^6−^ in the electro-assisted reduction of CO_2_ (Figure 11) [53]. The [α-SiW_11_O_39_Co]^6−^ contained a Co^II^ in place of a W^VI^. The square-pyramidal CoO_5_ with a vacant site was generated by losing a coordinated water molecule from CoO_5_(H_2_O) octahedral when the POM was extracted from aqueous to organic media. Except CO and HCHO, neither H_2_ nor other CO_2_ reduction products were detected. This indicated that the unique selectivity of [**α**-SiW_11_O_39_Co]^6−^ POM catalyst in the electroreduction of CO_2_. The turnover of CO_2_ to CO reached to 3.7 with the faradic efficiency of 13%. HCHO was the other detectable product. Its amount varied from 2.1 × 10^−7^ to 2.2 × 10^−6^ mol with the faradic yield varying from 25% (high HCHO content with low electrolysis charge) to 0.8% (low HCHO content with high electrolysis charge).

**Figure 12:**
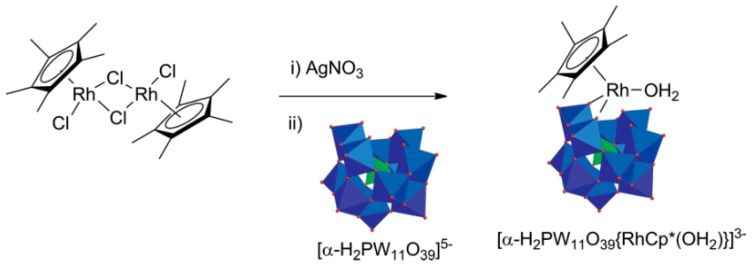
Synthesis of the organometallic derivative [α-H_2_PW_11_O_39_(Rh^III^Cp*(OH_2_))]^3−^. Color code: WO_6_ octahedron, navy blue; PO_4_ tetrahedron, green; O, red ball. Reprinted with permission from [54]. Copyright © 2019 Wiley-VCH Verlag GmbH and Co. KGaA.

Inspired by organometallic CO_2_ reduction catalyst [Cp*Rh^III^(bpy)Cl]^+^ (Cp* = pentamethyl-cyclopentadienyl anion; bpy = 2,2′-bipyridine), [α-H_2_PW_11_O_39_{Rh^III^Cp*(OH_2_)}]^3−^ with analogous coordination structure via grafting a {Cp*Rh^III^}^2+^ fragment on the monovacant [PW_11_O_39_]^7−^ anion was prepared (Figure 12) and tested as the electrocatalyst for CO_2_ reduction [54]. Compared to previously reported [CoSiW_11_O_39_]^6−^ catalyst, its electrochemical behavior in the presence of CO_2_ exhibited a clear improvement, strongly suggesting some interaction with the POM derivative despite the presence of a coordinating solvent. But H_2_ was still as major product (68% faradic yield) with HCOO^−^ as minor reduction product (4.5% faradic yield).

The remarkable capability of storing and donating electrons endows POMs the feature of reversibly transferring multi-electrons. Hence, selecting proper co-catalyst to establish synergistic catalytic systems containing POM catalyst is reasonable to achieve the efficient CO_2_ reduction. On one side, by taking the advantage of POMs on reversible transfer of multi-electrons or protons, co-catalysts can efficiently overcome the barrier of CO_2_ activation. On the other side, co-catalysts with good conductivity and adsorbing CO_2_ capability can facilitate the electroreduction and improve the activity of catalytic system. Poor electrical conductivity and electron-donating capability are the major drawbacks for MOFs being as efficient electrocatalysts. Therefore, the integrated use of POM and MOF would combine the strengths of each other and generate electrocatalysts with excellent activity [55,56,57].

**Figure 13:**
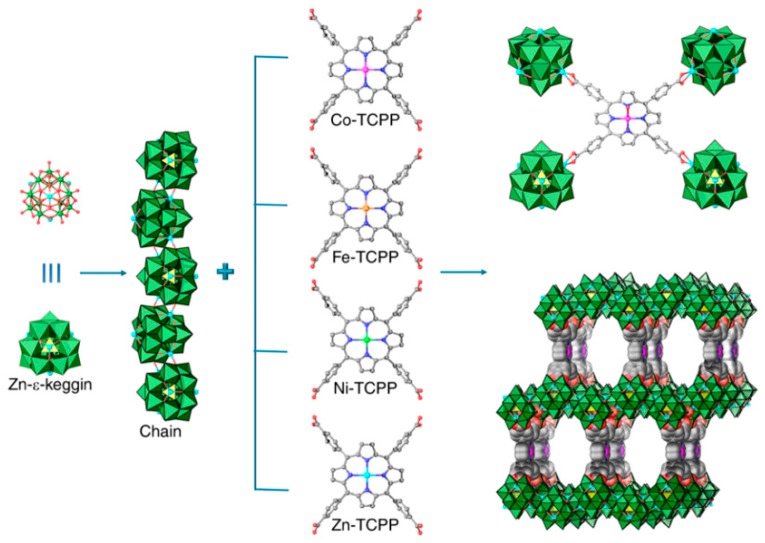
Schematic illustration of the structures of M-PMOFs (Polyoxometalate-Metalloporphyrin Organic Frameworks). Color code: MoO_6_ octahedra, bottle green; Zn, light blue ball; Ni, green ball; Fe, orange ball; Co, magenta ball, N, blue ball; C, grey ball; O, red ball; H was omitted. Reprinted with permission from [58]. Copyright © 2015 Attribution 4.0 International (CC BY 4.0).

Following the above intention, Lan et al. prepared various Polyoxometalate-Metalloporphyrin Organic Frameworks (PMOFs) by assembling Zn-ε-Keggin cluster ε-${\mathrm{PMo}}_{8}^{V}$ ${\mathrm{Mo}}_{4}^{\mathrm{VI}}$ O_40_Zn_4_ with M-TCPP (M-TCPP = tetrakis[4-carboxyphenyl]-porphyrinato-M; M = Co, Fe, Ni, Zn) via the links between Zn^2+^ ions of POM and carboxylates of porphyrin in hydrothermal conditions (Figure 13) and measured their catalytic performances (Table 6) [58]. In the skeleton of these M-PMOFs, the moieties of Zn-ε-Keggin cluster, as electron reservoirs, potently helped the electron transfer to CO_2_ reduction catalyst M-TCPP and improve its performance of CO_2_ reduction. The best results were obtained on Co-PMOF with lower onset potential, tafel slope, and electrochemical impedance as well as higher partial CO current density (j_CO_), electrochemical active surface area (ECSA), faradic efficiency for CO (FE_CO_) and turnover frequency (TOF)_._ Co-PMOF converted CO_2_ to CO with a superior faradic efficiency of 99%. The value of TOF was elevated to 1656 h^−1^ at −0.8 V. No evident activity attenuation was observed during the 36 h stability test, which indicated the robustness of Co-PMOF.

**Figure 14:**
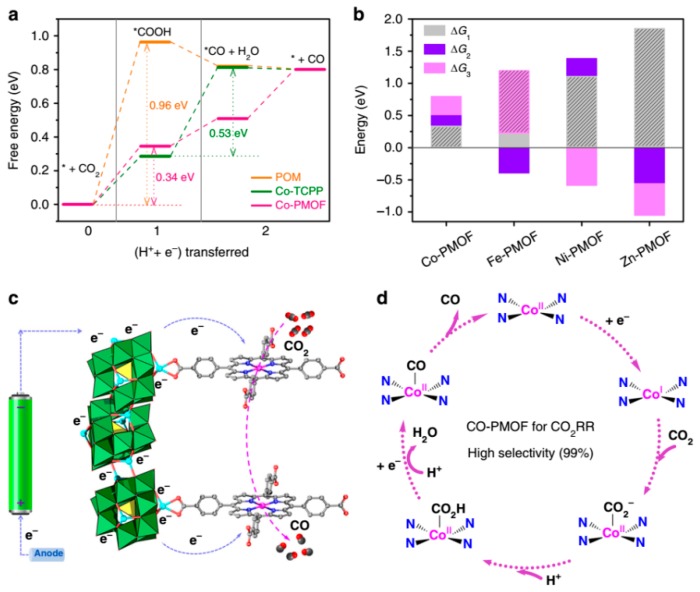
The DFT calculation and proposed reaction mechanism. The free energy diagrams CO_2_ reduction reaction (CO_2_RR) for the Zn-ε-Keggin POM, the Co-TCPP metalloporphyrin and the Co-PMOF (**a**); comparison of the free energy of each elementary reaction (ΔG_1_, ΔG_2_, and ΔG_3_ represent the free energy of * COOH formation, * CO formation, and CO desorption process, respectively) in CO_2_RR for Co-PMOF, Fe-PMOF, Ni-PMOF, and Zn-PMOF (**b**); proposed mechanistic scheme for the CO_2_RR on Co-PMOF (**c**,**d**); color code: MoO_6_ octahedra, bottle green; Zn, light blue ball; Co, magenta ball, N, blue ball; C, grey ball; O, red ball. Reprinted with permission from [58]. Copyright © 2015 Attribution 4.0 International (CC BY 4.0).

DFT calculations was employed to explicitly understand the excellent performance of Co-MPOF and synergism between Zn-ε-Keggin cluster and Co-TCPP. Owing to rather high Gibbs free energies of 0.96 eV (ΔG_1_ in Figure 14a), the formation of adsorbed intermediates * COOH was regarded as the rate-determining step (RDS) for CO_2_ reduction on Zn-ε-Keggin cluster. On the contrary, the formation of adsorbed intermediates * CO on Co-TCPP was RDS with high Gibbs free energies of 0.53 eV (ΔG_2_ in Figure 14a). In accordance with expectation, remarkable drops of both ΔG_1_ and ΔG_2_, particularly the much lower ΔG_1_ = 0.34 eV, were revealed. For Co-PMOF, the more favorable CO_2_ reduction active site is Co on Co-TCPP instead of Zn on POM. In fact, this Zn POM was inactive in CO_2_ reduction, and the synergistic effect was derived from the intramolecular electron transfer between the electron mediator of POM and Co-TCPP. The effect of different metal center in porphyrin was also computed (Figure 14b). The energy profile of reaction on different metal centers was well consistent with the outcomes of experiments. Based on the experimental results and theoretical calculations, the possible mechanism about reducing CO_2_ to CO on Co-PMOF was suggested. Firstly, the POM captures and transfers electrons from the electrode to the Co^II^ center. Subsequently, Co^II^ centers was reduced to Co^I^. Then Co^I^ interacts with CO_2_ to afford Co^II^-* COO^−^, which was converted to Co^II^-* CO via the proton-coupled electron transfer. Finally, CO is desorbed and released (Figure 14c,d).

The design and application of [α-SiW_12_O_40_]^4−^-modified AgNC@BSA catalyst (AgNC@BSA = silver nanoclusters capped with bovine serum albumin; BSA is both the stabilizer and reducing reagent for the synthesis of AgNC) in the electroreduction of CO_2_ to CO also exhibited the same concept of synergism [59]. Similar to the case of Co-PMOF, although [α-SiW_12_O_40_]^4−^ had little CO_2_ reduction activity, it played the role of electron transfer mediator and assisted the CO_2_ reduction through its strong interaction with CO_2_ on the surface of AgNC. It had excellent faradic efficiency (>75%) in DMF containing 1% (*v*/*v*) H_2_O. The overpotential of [α-SiW_12_O_40_]^4−^-modified AgNC@BSA electrode was about 0.7 V. The onset potential for this POM-decorated AgNC electrocatalyst was about 400 mV more positive than that on bulk Ag.

Metal nanoparticles stabilized by POMs was extensively researched [60,61]. By replacing BSA with POM as the stabilizer of AgNC, three Ag-POM nanocomposites respectively with [PMo_12_O_40_]^3−^, [α-SiW_12_O_40_]^4−^ and [PW_12_O_40_]^3−^ were synthesized by electrodeposition method [62]. POM is the promoter for the CO_2_ reduction. Due to the higher charge density and stronger basicity associated with [PMo_12_O_40_]^3−^, the activity of Ag-[PMo_12_O_40_]^3−^ nanocomposite was better than Ag-[PW_12_O_40_]^3−^ and Ag-[α-SiW_12_O_40_]^4−^. This nanocomposite exhibited efficient and sustained CO_2_ reduction at a wide potential range with faradic efficiency of 90 ± 5%. The Tafel slope, an inherent property of electrocatalyst determined by the rate-limiting step (formation of ${\mathrm{CO}}_{2}^{-}$ or protonation of ${\mathrm{CO}}_{2}^{-}$), calculated for Ag-[PMo_12_O_40_]^3−^ (60 mV dec^−1^) was very close to the theoretical value (59 mV dec^−1^), which indicated the faster formation of ${\mathrm{CO}}_{2}^{-}$ on the Ag-[PMo_12_O_40_]^3−^ nanocomposite-modified electrode

## 4. Electromicrobial Conversion of CO_2_

Since the introduction of lithoautotrophic bacterium *Ralstonia eutropha H16* as the production host for biological formate conversion as well as the coupling between this biological formate conversion method and the electroreductive conversion of CO_2_ to formate on indium cathode was employed to produce fuels for internal combustion engines from CO_2_ [63], the integration of electrocatalytic reduction with microbial conversion represents an edge-cutting strategy for the direct but non-photosynthetic CO_2_ conversion to important molecules.

Microorganisms, represented by *R. eutropha* and *Clostridium* spp, have been well harnessed to transform CO_2_ and H_2_ to energy-dense liquid fuels [64]. As potent hydrogen-oxidizing bacterium, *R. eutropha* has exhibited its versatility in synthesize poly[R-(−)-3-hydroxybutyrate], which is one of the ingredients to manufacture biodegradable plastics [65]. On the other aspect, many POMs-based catalysts have presented excellent activity for hydrogen evolution reaction (HER) [12,66]. Searching biocompatible POM-based HER catalysts and utilizing suitable microorganism to catalytically reduce CO_2_ by consuming H_2_ generated from HER can effectually accomplish the electromicrobial conversion of CO_2_.

**Figure 15:**
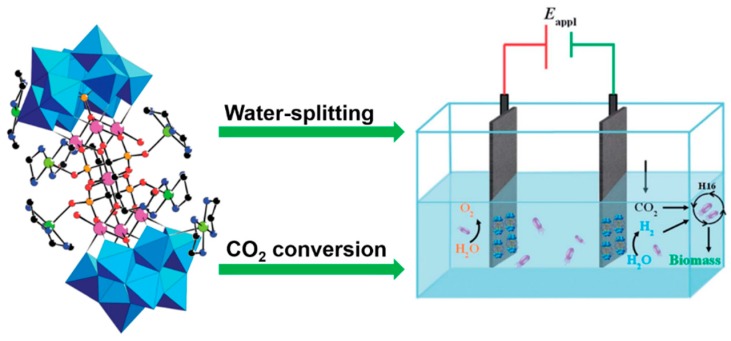
Schematic illustrations of the bioelectrochemical system for CO_2_ fixation. Color code: WO_6_ octahedra, light blue; Co, pink ball; P, orange ball; Cu spheres, green; C, black ball; N, dark blue ball; O, red ball; H atoms have been omitted for clarity. Reprinted with permission from [67]. Copyright © 2018 The Royal Society of Chemistry.

Recently, Zhang and co-workers demonstrated the feasibility of such reckoning. The combination of a heterometallic Co/Cu-containing polyoxometalate/carbon cloth (Cu_6_Co_7_/CC; Cu_6_Co_7_ = [Cu(en)_2_]_6_[((PW_9_O_34_)Co_3_(OH)(H_2_O)_2_(O_3_PC(O)(C_3_H_6_NH_3_)PO_3_))_2_Co]^2−^, en = ethanediamine; see the structure of Cu_6_Co_7_ POM in Figure 15; CC = carbon cloth) precatalyst with bacterium *R. eutropha H16* was developed to achieved the electricity-driven bioconversion of CO_2_ to biomass in neutral water [67]. Cu_6_Co_7_/CC hybrid material possessed good biocompatibility in this CO_2_ conversion system. It was evidenced that most of the H_2_ produced on the Cu_6_Co_7_/CC cathode was consumed by the bacteria for living and growth. In excess of half input electrical energy was transported into biomass. The authors assumed that the electricity would be supplied by a photovoltaic device with an efficiency of 18%, the expected overall solar-to-biomass efficiency could reach 10%, which would be nearly 10 times higher than the natural photosynthesis. This Cu_6_Co_7_/CC-*R. eutropha* hybrid system exemplified the potential to explore non-photosynthetic but highly efficient CO_2_-fixation methods by using POMs-based catalysts and solar electricity.

## 5. Non-Reductive CO_2_ Conversion to Carbonyl-Contained Organic Chemicals

**Scheme 2:**
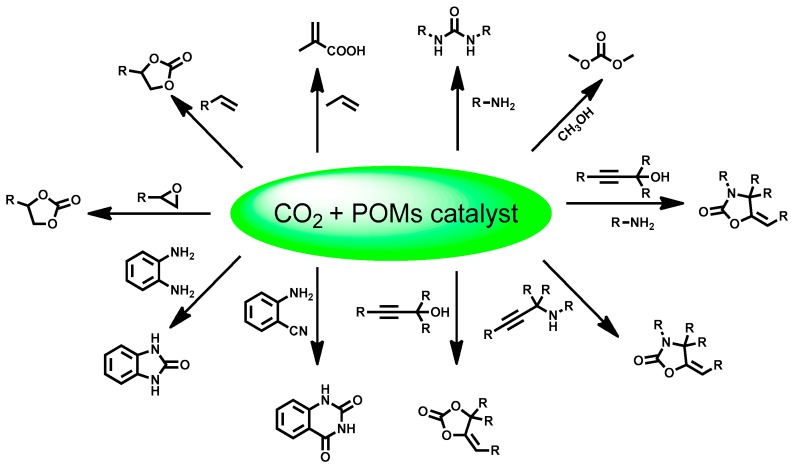
Non-reductive chemical fixation of CO_2_ by POM catalysts.

Non-reductive chemical CO_2_ conversion provides diverse alternatives to achieve both the synthesis of practical chemicals and CO_2_ fixation at the same time [68]. Currently, the industrial manufacture of several chemicals demands CO_2_ as starting material [69]. Predominantly, due to the basic properties of POM-based materials, the capture and transformation of CO_2_ with these materials has been widely explored. Therefore, the versatility of POMs-based materials in non-reductive chemical CO_2_ fixation has been fully presented. Various useful chemicals, including cyclic carbonate [19], dimethyl carbonate [70,71,72,73,74], urea [74], quinazoline-2,4-(1H,3H)-diones [74,75], 2-benzimidazolone [74], 2-oxazolidinones [18], α-methylene cyclic carbonate [74], and methacrylic acid [76,77] were able to be synthesized with POM-based catalyst (Scheme 2). Considering that several reviews have already given comprehensive summaries of representative progress on non-reductive CO_2_ fixation by POM catalysts [69,78,79], herein we briefly introduced the recent advances about non-reductive CO_2_ conversion to organic chemicals, which were not collected in the very recent review published in 2018 [79].

**Figure 16:**
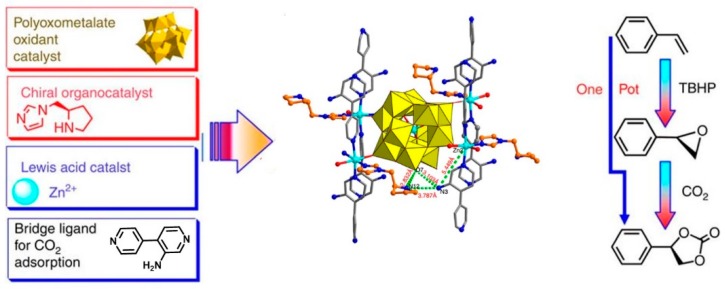
Synthetic procedure of the polyoxometallate-organocatalyst-metal organic frameworks (POMOFs) and the schematic representation of tandem catalysis for the asymmetric cyclic carbonate transformation from olefins and CO_2_. Color code: α-[ZnW_12_O_40_]^6−^, yellow polyhedral; Zn, cyan ball; N, blue ball; C, orange ball; O, red ball; H atom was omitted. Reprinted with permission from [81]. Copyright © 2015 Attribution 4.0 International (CC BY 4.0).

Various important applications of cyclic carbonates make the research on POM or other material-catalyzed synthesis of cyclic carbonates always receive long-lasting interests [80]. By integrating POM components, chiral organic catalysts and CO_2_ activator in the skeleton of a single MOF, the polyoxometallate-organocatalyst-metal organic framework (POMOF) was designed by Duan group [81]. Chiral cyclic carbonates were produced efficiently from olefins and CO_2_ on the POMOFs tandem catalyst. By ingeniously connecting Keggin-type POM anions α-[ZnW_12_O_40_]^6−^, l-proline-derived asymmetric organocatalysts pyrrolidine-2-yl-imidazole (PYI) and NH_2_-functionalized bridging links 2-amino-4,4′-bipyridine via the six-coordinated Zn^II^ nodes in these POMOFs (α-[ZnW_12_O_40_]^6−^-PYIs), a clear division of catalytic working for oxidation-coupled CO_2_ conversion was established that POM as an oxidation catalyst, pyrrolidine moiety as a chiral organocatalyst, Zn^II^ ions as Lewis acid catalyst to activate the epoxide intermediate and amino groups on 4,4′-bipyridines as renewable CO_2_ absorption reagent in this tandem process (Figure 16). The multi-catalytic sites were orderly distributed and spatially matched in the framework. The captured CO_2_ molecules are synergistically fixed and activated by well-positioned pyrrolidine and amine groups, providing further compatibility with the terminal W=O activated epoxidation intermediate and driving the tandem catalytic process in a single workup stage and an asymmetric fashion.

**Figure 17:**
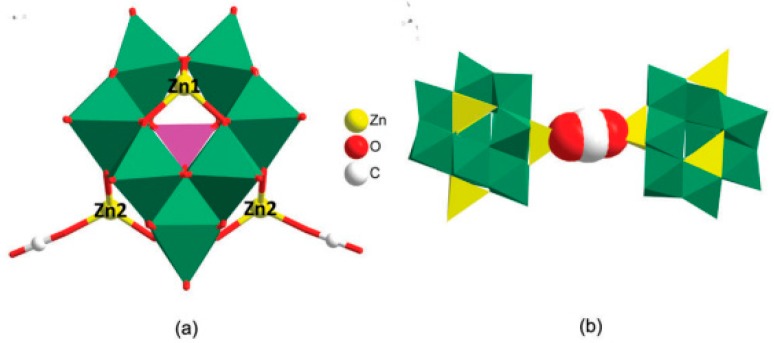
The basic building blocks (**a**) and coordination model of CO_2_ (**b**) in [PMo_12_O_40_Zn_4_(CO_2_)]^2−^. Color code: Zn, yellow ball; O, red ball; C, white ball; MoO_6_ octahedra, green; PO_4_ tetrahedron, magenta. Reprinted with permission from [19]. © 2018 The Royal Society of Chemistry.

In conjunction with ionic liquid co-catalyst, two kinds of POMs attached with metal carbonyl {P_2_W_15_O_56_Co_3_(H_2_O)_3_(OH)_3_Mn(CO)_3_}^8−^ [82] and {(Se_2_W_11_O_43_)(Mn(CO)_3_)_4_}^8−^ [83] were reported as efficient catalysts for the cycloaddition of CO_2_ with epoxides under mild conditions. A rare three-dimensional CO_2_-linked POM polymer {PMo_12_O_40_Zn_4_(CO_2_)}^2−^ exhibited superior performance in the cycloaddition of CO_2_ with epoxides [19]. The structural feature of catalyst is that the CO_2_ ligand connects with two Zn-ε-Keggin cores in a linear and symmetrical μ_2_-η^2^o,o coordination pattern(Figure 17). Polyoxoniobates (DBUH)_3_(NbO_5_), (TBA)_6_[Nb_10_O_28_] and Na_16_[SiNb_12_O_40_] were applied as Lewis base-type catalysts for the cycloaddition of CO_2_ with epoxides under halide-free conditions [84,85,86]. Carbon nanotubes-supported Fe_1.5_PMo_12_O_40_ (theoretical formulas) obtained 57.7% propylene oxide conversion and 99.0% propylene carbonate selectivity, both activity and selectivity were higher than Fe_1.5_PMo_12_O_40_, Co_1.5_PMo_12_O_40_, Cu_1.5_PMo_12_O_40_, and Zn_1.5_PMo_12_O_40_ (theoretical formulas). The good activity can be attributed to the well dispersion of the Fe_1.5_PMo_12_O_40_ on the CNTs [87].

## 6. Outlook

**Figure 18:**
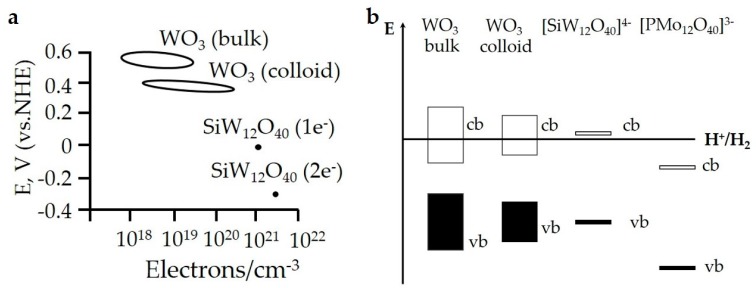
The effect of particle size on the flat band redox potentials (**a**) and schematic band structure diagram of POMs and WO_3_ (**b**). Reprinted with permission from [88]. © 1997 2018 Elsevier BV.

POMs are regarded as discrete metal oxide clusters with much smaller nanometric size than bulk metal oxides, therefore most of the distinctions on chemical properties, especially CO_2_ reductive conversion-related redox property, between bulk metal oxides and POMs were originated from quantum size effect. Quantum size effect leads to the changes of electronic configurations. These changes arise through systematic transformations in the density of electronic energy levels as a function of the size. By plotting the correlation between redox potential and electron density per volume (Figure 18a) as well as the diagram of band structure (Figure 18b), the quantum size effect-resulted distinction on redox property among bulk WO_3_, colloid WO_3_ and [SiW_12_O_40_]^4−^ has been clearly illustrated [88]. Despite the prevalence of both POMs and bulk oxides as photocatalysts of harvesting light in recent years, the smaller size endows POMs with wider gap and lower reduction potentials. The case given by Hill and Geletii et al. involving water oxidation catalyst [Co_4_(H_2_O)_2_(PW_9_O_34_)_2_]^10−^ (Co_4_POM) also manifested the distinction between POM and bulk metal oxide on redox property. In their report, (PW_9_O_34_)^9−^-sandwiched Co_4_ unit in Co_4_POM was unambiguously identified as the dominant active center for water splitting and not CoO_x_. [89]

The prospering arising of new POM structures provides vast opportunities to excavate latent catalysts for chemical CO_2_ conversion. Titanium oxo-clusters [90] and polyoxo-noble-metalates [91] are exemplified as two promising representatives among these new POM structures. Regarding titanium oxo-clusters (also named polyoxotitanates), there are diverse variants including titanium oxoalkoxides clusters Ti_n_O_m_(OR)_4n–2m_, titanium oxo-carboxo-alkoxides clusters Ti_n_O_2n–x/2–y/2_(OR)_x_(OOCR’)_y_, and titanium oxocarboxo clusters [Ti_n_O_m_(OOCR)_p_] (2m + p = 4n) [90]. The tunable wide polynuclearity (the number of titanium atoms n varies from 2 to 28) and facile fabrication of hybrid architectures with organic components by covalent bonding are the two major features of these clusters. MOF MIL-125 (MIL stands for Material from Insitut Lavoisier) consisting of 1,4-benzenedicarboxylate-connected titanium-oxo-hydroxo clusters has been reported to possess possible photocatalytic property when alcohols are adsorbed inside its framework [92]. Based on this indication and as the extensive applications of titanium oxide in CO_2_ photocatalytic reduction [93,94,95], there are likely to be extensive catalytic application of hybrid materials containing titanium oxo-cluster cores in CO_2_ reductive conversion. The emergance of various novel polyoxo-noble-metalates including polyoxopalladates, -platinates, and -aurates [91] also provides different prospects for catalytic CO_2_ reductive conversion. On these polyoxo-noble-metalates, the noble metal atoms Pd, Pt, and Au act as “addenda” atoms rather than as heteroatoms [91]. Despite that there are no direct accessible active sites on noble metal centers with saturated coordination environments, the very recent report about the catalytic Suzuki-Miyaura cross-coupling application of polyoxopalladate-based MOF materials has implied that these polyoxo-noble-metalates may acquire catalytic activity via in situ partial reduction [96]. For polyoxoplatinate [Pt_12_O_8_(SO_4_)_12_]^4−^ [97], although it is insoluble in any media, its six dumbbell-shaped [Pt_2_]^6+^ anionic units perhaps will attract electrocatalytic research interests, because they may be in-situ partially reduced to molecular mixed-valent metal clusters or platinum black-type “suboxides” [98]. Then these plausible in-situ generated species may be applied as HER catalysts for electromicrobial conversion of CO_2_.

More than the mode of LMCT, developing POM-based catalysts with the intrinsic function of metal-to-metal charge transfer (MMCT) will also be beneficial to look for more efficient POM-based approach to harvest visible-near infrared emission of sunlight with higher quantum yield. The MMCT transition occurs when two metal centers with different valence states are coupled by bridging ligands (M^a+^-O-M’^b+^). The two metal centers are respectively reduced and oxidized (M^a+^-O-M’^b+^ → M^(a−1)+^-O-M’^(b+1)+^) during this transition. The MMCT transition often takes place in polynuclear complexes [99]. The oxo-bridged and all-inorganic heterobinuclear units such as Zr^IV^-O-Co^II^ [100,101,102,103], Zr^IV^-O-Cu^I^ [104], Ti^IV^-O-Co^II^ [100], and Ti^IV^-O-Mn^II^ [105] supported on silica material that display MMCT transitions can extend the optical absorption from UV to visible regions. Some of these materials have been applied as catalyst or cocatalyst for CO_2_ photoreduction [101,102,103,104]. Several reported POM-based systems, such as the anchored polynuclear charge-transfer complexes consisting of Ce^III^ ions and Cu^II^-substituted Keggin-type Cu^II^PW_11_O_39_ [106], the metal-oxide nanoclusters consisting of Ce^III^ or Co^II^ ions and Keggin-type PW_12_O_40_^3−^ [107], [Co^II^W_12_O_40_]^6−^ [108], and [Co^II^(M^x^OH_y_)W_11_O_39_]^(12-x-y)−^ (M^x^OH_y_ = V^IV^O, Cr^III^(OH_2_), Mn^II^(OH_2_), Fe^III^(OH_2_), Co^II^(OH_2_), Ni^II^(OH_2_), Cu^II^(OH_2_), Zn^II^(OH_2_)) [109] have sufficiently demonstrated the feasibility that such molecular inorganic MMCT (or MPCT, metal-to-polyoxometalate charge transfer) [108,109] transition enable POM-based catalyst to function as efficient visible-light-driven multielectron-transfer catalysts. Regarding the metal-oxide nanoclusters consisting of Ce^III^ or Co^II^ ions and Keggin-type PW_12_O_40_^3−^, even the straightforward video guide of preparing catalytic material has been given [110]. These will further encourage to utilize MMCT of POM-based catalytic materials in CO_2_ reduction. It is noteworthy that prolonging the picosecond-scale lifetimes of photogenerated states during MMCT transition is still an important issue to be tackled [111]. The works from Lan and Hill et al. has validated the effects of heterometal location [108] and different addenda substitution [109] on the lifetime of photo-excited state.

On the other hand, the tools of advanced operando spectral characterization techniques [112], electrochemical measuring methods [113] and theoretical calculations [114], imbibing more understandings and attaining insights on catalytic mechanisms and structure–reactivity relationships of those reported POM-based catalysts will be helpful to design better POM catalysts for CO_2_ conversion with explicit destination. On CO_2_ photo/electroreduction, the hybridation of POM with various novel materials can provide chances to fabricate catalysts with broader visible-light absorption spectrum region or better selectivity but lower overpotential. In those systems with hybrid electro- or photocaytalytic CO_2_ catalysts, once the light-harvesting/electron-storage centers and catalytically active sites are designated in an integrated system, the key issue is the bridging of the two components—charge kinetics. Exploiting time-resolved spectroscopic techniques to characterize charge kinetics is useful to understand these hybrid catalysts and thus helps to improve their catalytic efficiencies [99].

In catalytic oxidation, POMs were looked upon as inorganic analogs of porphyrin [115,116,117]. The thriving development of transition metal porphyrin or phthalocyanine derivatives as heterogeneous molecular catalysts for electrochemical CO_2_ reduction [118] makes us have such confidence that this analogy can be still established in electrochemical CO_2_ reduction. Therefore, the accumulated understandings about electrochemical CO_2_ reduction by transition metal porphyrin or phthalocyanine may help the development of POM-based catalysts for the electroreductive CO_2_ conversion. By sunlight or solar electricity, producing fuels and chemicals via CO_2_ conversion is a promising and reliable option for largely reducing fossil fuel consumption in the future [119]. Thus POM-catalyzed transformation of CO_2_ with photo- or electrochemical methods will retain long-lasting interest of both academic and industrial research.

To the non-reductive CO_2_ conversion, searching for POMs with stronger Lewis basicity will benefit the elevation of turnover efficiency and the mitigation of harsh conditions under halogen-free condition. To enhance Lewis basicity, high negative charge density on the terminal oxygen atoms on POMs is required. Calculating and comparing natural bond orbital (NBO) charges of oxygen atoms in POMs has been demonstrated as a useful access to gain more insights into the Lewis basicity on POMs [84,120]. Making full use of both structural traits and diversity of POMs would help to break through some ceilings of practical CO_2_ chemical conversion.

## Figures and Tables

**Table 1:** Redox potentials for CO_2_ reduction.

Products	Products Reaction E^0^ (V)	E^0^ (V) pH = 7
-	HO + 2 h^+^ → 1/2O_2_ + 2H^+^	+0.82
-	CO_2_ + e^−^ → ${\mathrm{CO}}_{2}^{-}$	−1.9
HCOOH	CO_2_ + 2 H^+^ + 2 e^−^ → HCOOH	−0.61
CO	CO_2_ + 2 H^+^ + 2 e^−^ → CO + H_2_O	−0.53
HCHO	CO_2_ + 2 H^+^ + 4 e^−^ → HCHO + H_2_O	−0.48
CH_3_OH	CO_2_ + 6 H^+^ + 6 e^−^ → CH_3_OH + H_2_O	−0.38
CH_4_	CO_2_ + 8 H^+^ + 8 e^−^ → CH_4_ + 2H_2_O	−0.24
-	2 H^+^ + 2 e^−^ → H_2_	–0.41

**Table 2:** Catalytic performance of CO, H_2_, and CH_4_ from CO_2_ photoreduction ^a.^

Entry	Catalysts	Products (μmol g^−1^ h^−1^)		
		CO	CH_4_	H_2_
1	Au@NENU-10	12.8	2.1	2.6
2	Au@NENU-3	0.5	-	0.15
3	Au/Na_3_PW_12_ O_40_)	-	-	0.45
4	Au/K_7_(PTi_2_W_10_O_40_)	2.1	0.35	0.29
5	NENU-10	-	-	-
6	NENU-3	-	-	-
7	HKUST-1	-	-	-

^a.^ Reaction conditions: 100 mL quartz reactor, 15 mL H_2_O, 100 mg catalyst, 40 °C, 5 h, 300 W Xe lamp (λ > 420 nm). Solid catalyst was in the atmosphere of water vapor and CO_2_ instead of being immersed in water.

**Table 3:** The photocatalytic performance of the different catalysts ^a.^

Catalyst	HCOOH Yield (μmol)	TON	TOF (s^−1^)
{Mo_154_}_1165_	116.7	778	377
{Mn_6_P_3_W_24_}_931_	40.6	270	56
{Mo_132_}_1064_@RGO	205	1366	610

^a.^ Catalyst containing 0.15 μmol of {Mo_154_}, {Mn_6_P_3_W_24_} or {Mo_132_} units. The reaction mixtures were kept in a photo-reactor under UV-light lamp with 373 nm wavelength and 19 mW cm^−2^ energy density.

**Table 4:** The photocatalytic reduction of CO_2_ to CH_4_ using different catalysts ^a.^

Entry	Catalyst	CO (nmol/g)	CH_4_ (nmol/g)	CH_4_-TON (10^−3^)	CH_4_-TOF (10^−3^ h^−1^)	All-TON (10^−3^)	All-TOF (10^−3^ h^−1^)
1	NENU-605	52	170	104.1	5.5	135.9	7.2
2	NENU-606	68	402	241.4	10.5	282.2	12.3
3	NENU-607	47	70	15.2	0.75	25.4	1.3
4 ^b^	NENU-606	n.d.	n.d.	-	-	-	-
5 ^c^	NENU-606	n.d.	n.d.	-	-	-	-
6 ^d^	NENU-606	n.d.	n.d.	-	-	-	-
7	blank	n.d.	n.d.	-	-	-	-

^a.^ Reaction conditions: PS = [Ru(bpy)_3_]Cl_2_.6H_2_O (0.01 mmol, ) H_2_O (28 mL), SD = TEOA (2 mL), CO_2_ (1 atm), λ ≥420 nm, 20 °C; ^b^. without PS; ^c^. In dark; ^d^. Altering H_2_O with dry MeCN. n.d. = not detected.

**Table 5:** CO_2_ reduction potentials vs. SHE ^a^.

Products	Half-Electrochemical Thermodynamic Reactions	V vs. SHE
C	CO_2_(g) + 4 H^+^ + 4 e^−^ = C(s) + 2 H_2_O(l)	−0.210
C	CO_2_(g) + 2 H_2_O(l) + 4 e^−^ = C(s) + 4 OH^−^	−0.627
HCOOH	CO_2_(g) + 2 H^+^ + 2e^−^ = HCOOH(l)	−0.250
HCOO^−^	CO_2_(g) + 2 H_2_O(l) + 2 e^−^ = HCOO^−^ (aq) + OH^−^	−1.078
CO	CO_2_(g) + 2 H^+^ + 2 e^−^ = CO(g) + H_2_O(l)	−0.106
CO	CO_2_(g) + 2 H_2_O(l) + 2 e^−^ = CO(g) + 2 OH^−^	−0.934
HCHO	CO_2_(g) + 4 H^+^ + 4e^−^ = HCHO(l) + H_2_O(l)	−0.070
HCHO	CO_2_(g) + 3 H_2_O(l) + 4 e^−^ = HCHO(l) + 4 OH^−^	−0.898
CH_3_OH	CO_2_(g) + 6 H^+^ + 6 e^−^ = CH_3_OH(l) + H_2_O(l)	0.016
CH_3_OH	CO_2_(g) + 5 H_2_O(l) + 6 e^−^ = CH_3_OH(l) + 6 OH^−^	−0.812
CH_4_	CO_2_(g) + 8 H^+^ + 8 e^−^ = CH_4_(g) + 2 H_2_O (l)	0.169
CH_4_	CO_2_(g) + 6 H_2_O(l) + 8 e^−^ = CH_4_(g) + 8 OH^−^	−0.659
oxalic acid	2 CO_2_(g) + 2 H^+^ + 2 e^−^ = (COOH)_2_(aq)	−0.500
oxalate	2 CO_2_(g) + 2 e^−^ = C_2_$O_{4}^{2-}$(aq)	−0.590
ethylene	2 CO_2_(g) + 12 H^+^ + 12 e^−^ = CH_2_CH_2_(g) + 4 H_2_O(l)	0.064
ethylene	2 CO_2_(g) + 8 H_2_O(l) + 12 e^−^ = CH_2_CH_2_(g) + 12 OH^−^	−0.764
ethanol	2 CO_2_(g) + 12 H^+^ + 12 e^−^ = CH_3_CH_2_OH(l) + 3 H_2_O(l)	0.084
ethanol	2 CO_2_(g) + 9 H_2_O(l) + 12 e^−^ = CH_3_CH_2_OH(l) + 12 OH^−^	−0.744

^a.^ Reprinted with permission from [51]. Copyright © 2014 Royal Society of Chemistry.

**Table 6:** CO_2_ electroreduction performances ^a.^

Catalysts	E V vs. SHE	Onset Potential V	j_CO_ mA cm^−2^	Tafel Slope (mV dec^−1^)	ECSA mF cm^−2^	EIS Ω	FEco %	TOF H^−1^
Co-PMOF	−0.8	−0.35	18.08	98	12.17	9.83	98.7	1656
Fe-PMOF	−0.7	−0.53	0.47	211	10.26	10.26	28.8	17.45
Ni-PMOF	−0.8	−0.58	0.27	675	10.16	10.70	18.5	8.11
Zn-PMOF	−0.9	−0.60	0.02	206	9.83	12.17	0.95	0.005
Co-TMCP	−0.9	−0.53	NA ^b^	151	NA	NA	40	NA
TMCP	−0.6	−0.67	NA	552	NA	NA	0.77	NA
NNU-12	−0.6	−0.6	NA	413	NA	NA	1.8	NA

^a.^ Electrolyte and pH 0.5 M KHCO_3_, pH = 7.2; ^b^. NA means not mentioned in the [58].

## References

[1] Le Quéré C., Andrew R.M., Friedlingstein P., Sitch S., Hauck J., Pongratz J., Pickers P.A., Korsbakken J.I., Peters G.P., Canadell J.G. (2018). Global carbon budget 2018. Earth. Syst. Sci. Data.

[2] Sakakura T., Choi J.-C., Yasuda H. (2007). Transformation of carbon dioxide. Chem. Rev..

[3] Berzelius J. (1826). The preparation of the phosphomolybdate ion [PMo_12_O_40_]^3−^. Pogg. Ann..

[4] Song Y.-F. (2018). Polyoxometalate-Based Assemblies and Functional Materials.

[5] Van Eldik R., Cronin L. (2017). Polyoxometalate Chemistry.

[6] Liu S., Tang Z. (2010). Polyoxometalate-based functional nanostructured films: Current progress and future prospects. Nano Today.

[7] Zhang J., Huang Y., Li G., Wei Y. (2019). Recent advances in alkoxylation chemistry of polyoxometalates: From synthetic strategies, structural overviews to functional applications. Coord. Chem. Rev..

[8] Chen W., Wang E. (2013). Polyoxometalate Chemistry.

[9] Sartzi H., Miras H.N., Vila-Nadal L., Long D.L., Cronin L. (2015). Trapping the delta isomer of the polyoxometalate-based Keggin cluster with a tripodal ligand. Angew. Chem. Int. Ed..

[10] Bassil B.S., Kortz U. (2010). Recent advances in lanthanide-containing polyoxotungstates. Z. Anorg. Allg. Chem..

[11] Guo Y.-H., Hu C.-W. (2003). Porous hybrid photocatalysts based on polyoxometalates. J. Clust. Sci..

[12] Wang S.-S., Yang G.-Y. (2015). Recent advances in polyoxometalate-catalyzed reactions. Chem. Rev..

[13] Müller C.E., Iqbal J., Baqi Y., Zimmermann H., Röllich A., Stephan H. (2006). Polyoxometalates—a new class of potent ecto-nucleoside triphosphate diphosphohydrolase (NTPDase) inhibitors. Bioor. Med. Chem. Lett..

[14] Proust A., Thouvenot R., Gouzerh P. (2008). Functionalization of polyoxometalates: towards advanced applications in catalysis and materials science. Chem. Commun..

[15] Chen Q., Shen C., He L. (2018). Recent advances of polyoxometalate-catalyzed selective oxidation based on structural classification. Acta Crystallogr. Sect. C Struct. Chem..

[16] Szczepankiewicz S.H., Ippolito C.M., Santora B.P., Van De Ven T.J., Ippolito G.A., Fronckowiak L., Wiatrowski F., Power T., Kozik M. (1998). Interaction of carbon dioxide with transition-metal-substituted heteropolyanions in nonpolar solvents. spectroscopic evidence for complex formation. Inorg. Chem..

[17] Gao G., Li F., Xu L., Liu X., Yang Y. (2008). CO_2_ coordination by inorganic polyoxoanion in water. J. Am. Chem. Soc..

[18] He L.-N., Wang M.-Y., Song Q.-W., Ma R., Xie J.-N. (2016). Efficient conversion of carbon dioxide at atmospheric pressure to 2-oxazolidinones promoted by bifunctional Cu(II)-substituted polyoxometalate-based ionic liquids. Green Chem..

[19] Cheng W., Xue Y.-S., Luo X.-M., Xu Y., Xue Y. (2018). A rare three-dimensional POM-based inorganic metal polymer bonded by CO_2_ with high catalytic performance for CO_2_ cycloaddition. Chem. Commun..

[20] Fang X., Anderson T.M., Neiwert W.A., Hill C.L. (1998). Yttrium polyoxometalates. Synthesis and characterization of a carbonate-encapsulated sandwich-type complex. Inorg. Chem..

[21] Chen B., Neumann R. (2018). Coordination of carbon dioxide to the Lewis acid site of a zinc-substituted polyoxometalate and formation of an adduct using a polyoxometalate-2,4,6-trimethylpyridine frustrated Lewis pair. Eur. J. Inorg. Chem..

[22] Garai S., Haupt E.T.K., Bögge H., Merca A., Müller A. (2012). Picking up 30 CO_2_ molecules by a porous metal oxide capsule based on the same number of receptors. Angew. Chem. Int. Ed..

[23] Selimkhanov J., Taylor B., Yao J., Pilko A., Albeck J., Hoffmann A., Tsimring L., Wollman R. (2014). Accurate information transmission through dynamic biochemical signaling networks. Science.

[24] Li K., Peng B., Peng T. (2016). Recent advances in heterogeneous photocatalytic CO_2_ conversion to solar fuels. ACS Catal..

[25] Sohn Y., Huang W., Taghipour F. (2017). Recent progress and perspectives in the photocatalytic CO_2_ reduction of Ti-oxide-based nanomaterials. Appl. Surf. Sci..

[26] Inoue T., Fujishima A., Konishi S., Honda K. (1979). Photoelectrocatalytic reduction of carbon dioxide in aqueous suspensions of semiconductor powders. Nature.

[27] Zeng S., Kar P., Thakur U.K., Shankar K. (2018). A review on photocatalytic CO_2_ reduction using perovskite oxide nanomaterials. Nanotechnology.

[28] Shi R., Waterhouse G.I., Zhang T. (2017). Recent Progress in Photocatalytic CO_2_ reduction over perovskite oxides. Sol. RRL.

[29] Ye S., Wang R., Wu M.-Z., Yuan Y.-P. (2015). A review on *g*-C_3_N_4_ for photocatalytic water splitting and CO_2_ reduction. Appl. Surf. Sci..

[30] Li R., Zhang W., Zhou K. (2018). Metal-organic-framework-based catalysts for photoreduction of CO_2_. Adv. Mater..

[31] Sprick R.S., Jiang J.-X., Bonillo B., Ren S., Ratvijitvech T., Guiglion P., Zwijnenburg M.A., Adams D.J., Cooper A.I. (2015). Tunable organic photocatalysts for visible-light-driven hydrogen evolution. J. Am. Chem. Soc..

[32] Ghosh S., Kouamé N.A., Ramos L., Rémita S., Dazzi A., Deniset-Besseau A., Beaunier P., Goubard F., Aubert P.-H., Remita H. (2015). Conducting polymer nanostructures for photocatalysis under visible light. Nat. Mater..

[33] Papaconstantinou E. (1989). Photochemistry of polyoxometallates of molybdenum and tungsten and/or vanadium. Chem. Soc. Rev..

[34] Hill C.L., Prosser-McCartha C.M. (1993). Photocatalytic and Photoredox Properties of Polyoxometalate Systems.

[35] Nemodruk A.A., Bezrogova E.V. (1969). The use of photochemical reduction for the determination of silicon and phosphorus as their blue heteropoly acids. Zh. Anal. Khim..

[36] Morosanova S.A., Kolli N.Y., Kushnirenko T.G. (1977). Photochemical reduction of 12-molybdoarsenic acid in aqueous-organic media. Zh. Anal. Khim..

[37] Streb C. (2012). New trends in polyoxometalate photoredox chemistry: From photosensitisation to water oxidation catalysis. Dalton Trans..

[38] Tucher J., Wu Y., Nye L.C., Ivanović-Burmazović I., Khusniyarov M.M., Streb C. (2012). Metal substitution in a Lindqvist polyoxometalate leads to improved photocatalytic performance. Dalton Trans..

[39] Walsh J.J., Bond A.M., Forster R.J., Keyes T.E. (2016). Hybrid polyoxometalate materials for photo(electro-) chemical applications. Coord. Chem. Rev..

[40] Khenkin A.M., Efremenko I., Weiner L., Martin J.M.L., Neumann R. (2010). Photochemical reduction of carbon dioxide catalyzed by a ruthenium-substituted polyoxometalate. Chem. Eur. J..

[41] Ettedgui J., Diskin-Posner Y., Weiner L., Neumann R. (2011). Photoreduction of carbon dioxide to carbon monoxide with hydrogen catalyzed by a rhenium(I) phenanthroline−polyoxometalate hybrid complex. J. Am. Chem. Soc..

[42] Poblet J.M. (2016). The Photoreduction mechanism of CO_2_ to CO catalyzed by a rhenium(I)-polyoxometalate hybrid compound. ACS Catal..

[43] Haviv E., Shimon L.J.W., Neumann R. (2017). Photochemical reduction of CO_2_ with visible light using a polyoxometalate as photoreductant. Chem. Eur. J..

[44] Zhou J., Chen W., Sun C., Han L., Qin C., Chen M., Wang X., Wang E., Su Z. (2017). Oxidative polyoxometalates modified graphitic carbon nitride for visible-light CO_2_ reduction. ACS Appl. Mater. Interfaces.

[45] Liu S.-M., Zhang Z., Li X., Jia H., Ren M., Liu S. (2018). Ti-substituted Keggin-type polyoxotungstate as proton and electron reservoir encaged into metal-organic framework for carbon dioxide photoreduction. Adv. Mater. Interfaces.

[46] Das S., Biswas S., Balaraju T., Barman S., Pochamoni R., Roy S. (2016). Photochemical reduction of carbon dioxide coupled with water oxidation using various soft-oxometalate (SOM) based catalytic systems. J. Mater. Chem. A.

[47] Das S., Kumar S., Garai S., Pochamoni R., Paul S., Roy S. (2017). Softoxometalate [{K_6.5_Cu(OH)(_8.5_)(H_2_O)(_7.5_)}(_0.5_)@{K_3_PW_12_O_40_}]_(n)_ (n=1348-2024) as an efficient inorganic material for CO_2_ reduction with concomitant water oxidation. ACS Appl. Mater. Interfaces.

[48] Das S., Balaraju T., Barman S., Sreejith S.S., Pochamoni R., Roy S. (2018). A Molecular CO_2_ reduction catalyst based on giant polyoxometalate {Mo-368}. Front. Chem..

[49] Yamase T., Sugeta M. (1990). Photoreduction of CO_2_ to CH_4_ in water using dititanodecatungstophosphate as multielectron transfer catalyst. Inorg. Chim. Acta..

[50] Xie S.-L., Liu J., Dong L.-Z., Li S.-L., Lan Y.-Q., Su Z.-M. (2019). Hetero-metallic active sites coupled with strongly reductive polyoxometalate for selective photocatalytic CO_2_-to-CH_4_ conversion in water. Chem. Sci..

[51] Qiao J., Liu Y., Hong F., Zhang J. (2014). A review of catalysts for the electroreduction of carbon dioxide to produce low-carbon fuels. Chem. Soc. Rev..

[52] Paul J., Page P., Sauers P., Ertel K., Pasternak C., Lin W., Kozik M. (2012). Transition-Metal-Substituted Heteropoly Anions in Nonpolar Solvents-Structures and Interaction with Carbon Dioxide.

[53] Girardi M., Blanchard S., Griveau S., Simon P., Fontecave M., Bedioui F., Proust A. (2015). Electro-assisted reduction of CO_2_ to CO and formaldehyde by (TOA)_6_[α-SiW_11_O_39_Co(_)] polyoxometalate. Eur. J. Inorg. Chem..

[54] Girardi M., Platzer D., Griveau S., Bedioui F., Alves S., Proust A., Blanchard S. (2019). Assessing the electrocatalytic properties of the {Cp*Rh^III^}^2+^-polyoxometalate derivative [H_2_PW_11_O_39_{(Rh^III^Cp*(OH_2_)}]^3−^ towards CO_2_ reduction. Eur. J. Inorg. Chem..

[55] Szczęśniak B., Choma J., Jaroniec M. (2018). Gas adsorption properties of hybrid graphene-MOF materials. J. Colloid Interface Sci..

[56] Genovese M., Lian K. (2015). Polyoxometalate modified inorganic–organic nanocomposite materials for energy storage applications: A review. Curr. Opin. Solid State Mater. Sci..

[57] Fan D., Hao J., Wei Q. (2012). Assembly of polyoxometalate-based composite materials. J. Inorg. Organomet. Polym. Mater..

[58] Wang Y.-R., Huang Q., He C.-T., Chen Y., Liu J., Shen F.-C., Lan Y.-Q. (2018). Oriented electron transmission in polyoxometalate-metalloporphyrin organic framework for highly selective electroreduction of CO_2_. Nat. Commun..

[59] Guo S.-X., MacFarlane D.R., Zhang J. (2016). Bioinspired electrocatalytic CO_2_ reduction by bovine serum albumin-capped silver nanoclusters mediated by [α-SiW_12_O_40_]^4−^. Chemsuschem.

[60] Wang Y., Weinstock I.A. (2012). Polyoxometalate-decorated nanoparticles. Chem. Soc. Rev..

[61] Jameel U., Zhu M., Chen X., Tong Z. (2016). Recent progress of synthesis and applications in polyoxometalate and nanogold hybrid materials. J. Mater. Sci..

[62] Guo S.-X., Li F., Chen L., Macfarlane D.R., Zhang J. (2018). Polyoxometalate-promoted electrocatalytic CO_2_ reduction at nanostructured silver in dimethylformamide. ACS Appl. Mater. Interfaces.

[63] Li H., Opgenorth P.H., Wernick D.G., Rogers S., Wu T.-Y., Higashide W., Malati P., Huo Y.-X., Cho K.M., Liao J.C. (2012). Integrated electromicrobial conversion of CO_2_ to higher alcohols. Science.

[64] Hawkins A.S., McTernan P.M., Lian H., Kelly R.M., Adams M.W. (2013). Biological conversion of carbon dioxide and hydrogen into liquid fuels and industrial chemicals. Curr. Opin. Biotechnol..

[65] Pohlmann A., Fricke W.F., Reinecke F., Kusian B., Liesegang H., Cramm R., Eitinger T., Ewering C., Pötter M., Schwartz E. (2006). Genome sequence of the bioplastic-producing “Knallgas” bacterium Ralstonia eutropha H16. Nat. Biotechnol..

[66] Freire C., Nunes M., Fernandes D.M., Abdelkader V.K. (2018). POM&MOF-based electrocatalysts for energy-related reactions. ChemCatChem.

[67] Wang M., Zhong W., Zhang S., Liu R., Xing J., Zhang G. (2018). An overall water-splitting polyoxometalate catalyst for the electromicrobial conversion of CO_2_ in neutral water. J. Mater. Chem. A.

[68] Yuan G., Qi C., Wu W., Jiang H. (2017). Recent advances in organic synthesis with CO_2_ as C1 synthon. Green Sustain. Chem..

[69] Yu B., Zou B., Hu C.-W. (2018). Recent applications of polyoxometalates in CO_2_ capture and transformation. J. CO_2_ Util..

[70] La K.W., Youn M.H., Chung J.S., Baeck S.H., Song I.K. (2007). Synthesis of dimethyl carbonate from methanol and carbon dioxide by heteropolyacid/metal oxide catalysts. Solid State Phenom..

[71] Aouissi A., Al-Othman Z.A., Al-Amro A. (2010). Gas-phase synthesis of dimethyl carbonate from methanol and carbon dioxide over Co_1.5_PW_12_O_40_ Keggin-type heteropolyanion. Int. J. Mol. Sci..

[72] Lee H.J., Park S., Jung J.C., Song I.K. (2011). Direct synthesis of dimethyl carbonate from methanol and carbon dioxide over H_3_PW_12_O_40_/Ce_X_Zr_1-X_O_2_ catalysts: Effect of acidity of the catalysts. Korean J. Chem. Eng..

[73] La K.W., Jung J.C., Kim H., Baeck S.-H., Song I.K. (2007). Effect of acid–base properties of H_3_PW_12_O_40_/Ce_x_Ti_1−x_O_2_ catalysts on the direct synthesis of dimethyl carbonate from methanol and carbon dioxide: A TPD study of H_3_PW_12_O_40_/Ce_x_Ti_1−x_O_2_ catalysts. J. Mol. Catal. A: Chem..

[74] Kimura T., Kamata K., Mizuno N. (2012). A bifunctional tungstate catalyst for chemical fixation of CO_2_ at atmospheric pressure. Angew. Chem. Int. Ed..

[75] Sunaba H., Kimura T., Kamata K., Mizuno N. (2012). Efficient [WO_4_]^2−^ -catalyzed chemical fixation of carbon dioxide with 2-aminobenzonitriles to quinazoline-2,4(1H,3H)-diones. Inorg. Chem..

[76] Wang D., Zhong S. (2003). Study on CuPMo/TiO_2_ catalyst for direct synthesis of methacrylic acid from propylene and carbon dioxide. Chin. J. Catal..

[77] Wang D.-W., Zhong S.-H. (2004). Study on CuPW/TiO_2_ catalyst for direct synthesis of MAA from propylene and carbon dioxide. J. Fuel Chem. Technol..

[78] Wang M.-Y., Ma R., He L.-N. (2016). Polyoxometalate-based ionic liquids-promoted CO_2_ conversion. Sci. China Chem..

[79] Kamata K., Sugahara K. (2017). Base catalysis by mono- and polyoxometalates. Catalysts.

[80] Guo L., Jin X., Wang X., Yin L., Wang Y., Yang Y.-W. (2018). Immobilizing polyether imidazole ionic liquids on zsm-5 zeolite for the catalytic synthesis of propylene carbonate from carbon dioxide. Molecules.

[81] Han Q., Qi B., Ren W., He C., Niu J., Duan C. (2015). Polyoxometalate-based homochiral metal-organic frameworks for tandem asymmetric transformation of cyclic carbonates from olefins. Nat. Commun..

[82] Jia J., Niu Y., Zhang P., Zhang D., Ma P., Zhang C., Niu J., Wang J. (2017). A Monomeric tricobalt(II)-substituted dawson-type polyoxometalate decorated by a metal carbonyl group: [P_2_W_15_O_56_Co_3_(H_2_O)_3_(OH)_3_Mn(CO)_3_]^8−^. Inorg. Chem..

[83] Lu J., Ma X., Singh V., Zhang Y., Wang P., Feng J., Ma P., Niu J., Wang J. (2018). Facile CO_2_ cycloaddition to epoxides by using a tetracarbonyl metal selenotungstate derivate [{Mn(CO)_3_}_4_(Se_2_W_11_O_43_)]^8−^. Inorg. Chem..

[84] Ge W., Wang X., Zhang L., Du L., Zhou Y., Wang J. (2016). Fully-occupied Keggin type polyoxometalate as solid base for catalyzing CO_2_ cycloaddition and Knoevenagel condensation. Catal. Sci. Technol..

[85] Chen A., Chen C., Xiu Y., Liu X., Chen J., Guo L., Zhang R., Hou Z. (2015). Niobate salts of organic base catalyzed chemical fixation of carbon dioxide with epoxides to form cyclic carbonates. Green Chem..

[86] Hayashi S., Yamazoe S., Koyasu K., Tsukuda T. (2017). Lewis base catalytic properties of [Nb_10_O_28_]^6−^ for CO_2_ fixation to epoxide: kinetic and theoretical studies. Chem. – Asian J..

[87] Al-Garni T., Al-Jallal N., Aouissi A. (2017). Synthesis of propylene carbonate from epoxide and CO_2_ catalyzed by carbon nanotubes supported Fe_1.5_PMo_12_O_40_. J. Chem..

[88] Gómez-Romer P. (1997). Polyoxometalates as photoelectrochemical models for quantum-sized colloidal semiconducting oxides. Solid State Ionics.

[89] Vickers J.W., Lv H., Sumliner J.M., Zhu G., Luo Z., Musaev D.G., Geletii Y.V., Hill C.L. (2013). Differentiating homogeneous and heterogeneous water oxidation catalysis: confirmation that [Co_4_(H_2_O)_2_(α-PW_9_O_34_)_2_]^10−^ is a molecular water oxidation catalyst. J. Am. Chem. Soc..

[90] Rozes L., Sanchez C. (2011). Titanium oxo-clusters: Precursors for a Lego-like construction of nanostructured hybrid materials. Chem. Soc. Rev..

[91] Izarova N.V., Pope M.T., Kortz U. (2012). Noble metals in polyoxometalates. Angew. Chem., Int. Ed..

[92] Dan-Hardi M., Serre C., Frot T., Rozes L., Maurin G., Sanchez C., Férey G. (2009). A new photoactive crystalline highly porous titanium(IV) dicarboxylate. J. Am. Chem. Soc..

[93] Habisreutinger S.N., Schmidt-Mende L., Stolarczyk J.K., Schmidt-Mende L., Schmidt-Mende L. (2013). Photocatalytic reduction of CO_2_ on TiO_2_ and other semiconductors. Angew. Chem. Int. Ed..

[94] Abdullah H., Khan M.M.R., Ong H.R., Yaakob Z. (2017). Modified TiO_2_ photocatalyst for CO_2_ photocatalytic reduction: an overview. J. CO_2_ Util..

[95] Li X., Yu J., Jaroniec M., Chen X. (2019). Cocatalysts for selective photoreduction of CO_2_ into solar fuels. Chem. Rev..

[96] Bhattacharya S., Ayass W.W., Taffa D.H., Schneemann A., Semrau A.L., Wannapaiboon S., Altmann P.J., Pöthig A., Nisar T., Balster T. (2019). Discovery of polyoxo-noble-metalate-based metal–organic frameworks. J. Am. Chem. Soc..

[97] Pley M., Wickleder M.S. (2004). The cluster ion [Pt_12_O_8_(SO_4_)12]^4−^. Angew. Chem. Int. Ed..

[98] Goloboy J.C., Klemperer W.G. (2009). Are particulate noble-metal catalysts metals, metal oxides, or something in-between?. Angew. Chem. Int. Ed..

[99] Gao C., Wang J., Xu H., Xiong Y. (2017). Coordination chemistry in the design of heterogeneous photocatalysts. Chem. Soc. Rev..

[100] Hill A.D., Katsoukis G., Frei H. (2018). Photoinduced electron transfer from ZrOCo binuclear light absorber to pyridine elucidated by transient optical and infrared spectroscopy. J. Phys. Chem. C.

[101] Kim W., Frei H. (2015). Directed assembly of cuprous oxide nanocatalyst for CO_2_ reduction coupled to heterobinuclear ZrOCo^II^ light absorber in mesoporous silica. ACS Catal..

[102] Kim W., Yuan G., McClure B.A., Frei H. (2014). Light induced carbon dioxide reduction by water at binuclear ZrOCo^II^ unit coupled to Ir oxide nanocluster catalyst. J. Am. Chem. Soc..

[103] Macnaughtan M.L., Soo H.S., Frei H. (2014). Binuclear ZrOCo metal-to-metal charge-transfer unit in mesoporous silica for light-driven CO_2_ reduction to CO and formate. J. Phys. Chem. C.

[104] Lin W., Frei H. (2005). Photochemical CO_2_ splitting by metal-to-metal charge-transfer excitation in mesoporous ZrCu(I)-MCM-41 silicate sieve. J. Am. Chem. Soc..

[105] McClure B.A., Frei H. (2014). Excited state electron transfer of all-inorganic heterobinuclear TiOMn^2+^ chromophore anchored on silica nanoparticle surface. J. Phys. Chem. C.

[106] Takashima T., Yamaguchi A., Hashimoto K., Nakamura R. (2012). Multielectron-transfer reactions at single Cu(II) centers embedded in polyoxotungstates driven by photo-induced metal-to-metal charge transfer from anchored Ce(III) to framework W(VI). Chem. Commun..

[107] Yamaguchi A., Takashima T., Hashimoto K., Nakamura R. (2017). Design of metal-to-metal charge-transfer chromophores for visible-light activation of oxygen-evolving Mn oxide catalysts in a polymer film. Chem. Mater..

[108] Glass E.N., Fielden J., Kaledin A.L., Musaev D.G., Lian T., Hill C.L. (2014). Extending metal-to-polyoxometalate charge transfer lifetimes: the effect of heterometal location. Chem. Eur. J..

[109] Glass E.N., Musaev D.G., Lian T., Hill C.L., Fielden J., Huang Z., Xiang X. (2016). Transition metal substitution effects on metal-to-polyoxometalate charge transfer. Inorg. Chem..

[110] Yamaguchi A., Takashima T., Hashimoto K., Nakamura R. (2018). Preparation of polyoxometalate-based photo-responsive membranes for the photo-activation of manganese oxide catalysts. J. Vis. Exp..

[111] Meng Y.-S., Sato O., Liu T. (2018). Manipulating metal-to-metal charge transfer for materials with switchable functionality. Angew. Chem. Int. Ed..

[112] Zhang Y., Fu D., Xu X., Sheng Y., Xu J., Han Y.-F. (2016). Application of operando spectroscopy on catalytic reactions. Curr. Opin. Chem. Eng..

[113] Freund T., Gomes W.P. (1970). Electrochemical methods for investigating catalysis by semiconductors. Catal. Rev..

[114] Lopez X., Carbó J.J., Bo C., Poblet J.M. (2012). Structure, properties and reactivity of polyoxometalates: A theoretical perspective. Chem. Soc. Rev..

[115] Lyon D.K., Miller W.K., Novet T., Domaille P.J., Evitt E., Johnson D.C., Finke R.G. (1991). Highly oxidation resistant inorganic-porphyrin analog polyoxometalate oxidation catalysts. 1. The synthesis and characterization of aqueous-soluble potassium salts of α_2_-P_2_W_17_O_61_(M^n+^·OH_2_)^(n-10)^ and organic solvent soluble tetra-n-butylammonium salts of α_2_-P_2_W_17_O_61_(M^n+^·Br)^(n-11)^ (M = Mn^3+^, Fe^3+^, Co^2+^, Ni^2+^, Cu^2+^). J. Am. Chem. Soc..

[116] Randell W.J., Weakley T.J.R., Finke R.G. (1993). Oxidation resistant inorganic porphyrin analog polyoxometalates. 3. The synthesis and X-ray crystallographic characterization of a new heteropolyoxoanion structural type, the diruthenium-oxo-bridged “bimetallic unorganic-polyphyrin analog KLi_15_[O{Ru^IV^Cl(α_2_-P_2_W_17_O_61_)}_2_]·2KCl·60H_2_O”. Inorg. Chem..

[117] Neumann R., Dahan M. (1997). A ruthenium-substituted polyoxometalate as an inorganic dioxygenase for activation of molecular oxygen. Nature.

[118] Corbin N., Zeng J., Williams K., Manthiram K. (2019). Heterogeneous molecular catalysts for electrocatalytic CO_2_ reduction. Nano Res..

[119] Shih C.F., Zhang T., Li J., Bai C. (2018). Powering the future with liquid sunshine. Joule.

[120] Xu Q., Niu Y., Wang G., Li Y., Zhao Y., Singh V., Niu J., Wang J. (2018). Polyoxoniobates as a superior Lewis base efficiently catalyzed Knoevenagel condensation. Mol. Catal..

